# Tau depletion in human neurons mitigates Aβ-driven toxicity

**DOI:** 10.1038/s41380-024-02463-2

**Published:** 2024-02-15

**Authors:** Bryan Ng, Jane Vowles, Féodora Bertherat, Ajantha Abey, Peter Kilfeather, Dayne Beccano-Kelly, M. Irina Stefana, Darragh P. O’Brien, Nora Bengoa-Vergniory, Phillippa J. Carling, John A. Todd, Tara M. Caffrey, Natalie Connor-Robson, Sally A. Cowley, Richard Wade-Martins

**Affiliations:** 1 Department of Physiology, Anatomy and Genetics, University of Oxford, South Parks Road, Oxford, OX1 3QU UK; 2 Kavli Institute for Nanoscience Discovery, Dorothy Crowfoot Hodgkin Building, University of Oxford, South Parks Road, Oxford, OX1 3QU UK; 3 James and Lillian Martin Centre for Stem Cell Research, Sir William Dunn School of Pathology, University of Oxford, South Parks Road, OX1 3RE Oxford, UK; 4 JDRF/Wellcome Diabetes and Inflammation Laboratory, Wellcome Centre for Human Genetics, Nuffield Department of Medicine, NIHR Oxford Biomedical Research Centre, University of Oxford, Oxford, OX3 7BN UK; 5 Target Discovery Institute, Centres for Medicines Discovery, Nuffield Department of Medicine, University of Oxford, NDM Research Building, Old Road Campus, Oxford, OX3 7FZ UK; 6 Oxford Drug Discovery Institute, Target Discovery Institute, University of Oxford, NDM Research Building, Old Road Campus, Oxford, OX3 7FZ UK

**Keywords:** Psychiatric disorders, Molecular biology, Stem cells, Neuroscience

## Abstract

Alzheimer’s disease (AD) is an age-related neurodegenerative condition and the most common type of dementia, characterised by pathological accumulation of extracellular plaques and intracellular neurofibrillary tangles that mainly consist of amyloid-β (Aβ) and hyperphosphorylated tau aggregates, respectively. Previous studies in mouse models with a targeted knock-out of the microtubule-associated protein tau *(Mapt)* gene demonstrated that Aβ-driven toxicity is tau-dependent. However, human cellular models with chronic tau lowering remain unexplored. In this study, we generated stable tau-depleted human induced pluripotent stem cell (iPSC) isogenic panels from two healthy individuals using CRISPR-Cas9 technology. We then differentiated these iPSCs into cortical neurons in vitro in co-culture with primary rat cortical astrocytes before conducting electrophysiological and imaging experiments for a wide range of disease-relevant phenotypes. Both AD brain derived and recombinant Aβ were used in this study to elicit toxic responses from the iPSC-derived cortical neurons. We showed that tau depletion in human iPSC-derived cortical neurons caused considerable reductions in neuronal activity without affecting synaptic density. We also observed neurite outgrowth impairments in two of the tau-depleted lines used. Finally, tau depletion protected neurons from adverse effects by mitigating the impact of exogenous Aβ-induced hyperactivity, deficits in retrograde axonal transport of mitochondria, and neurodegeneration. Our study established stable human iPSC isogenic panels with chronic tau depletion from two healthy individuals. Cortical neurons derived from these iPSC lines showed that tau is essential in Aβ-driven hyperactivity, axonal transport deficits, and neurodegeneration, consistent with studies conducted in *Mapt−/−* mouse models. These findings highlight the protective effects of chronic tau lowering strategies in AD pathogenesis and reinforce the potential in clinical settings. The tau-depleted human iPSC models can now be applied at scale to investigate the involvement of tau in disease-relevant pathways and cell types.

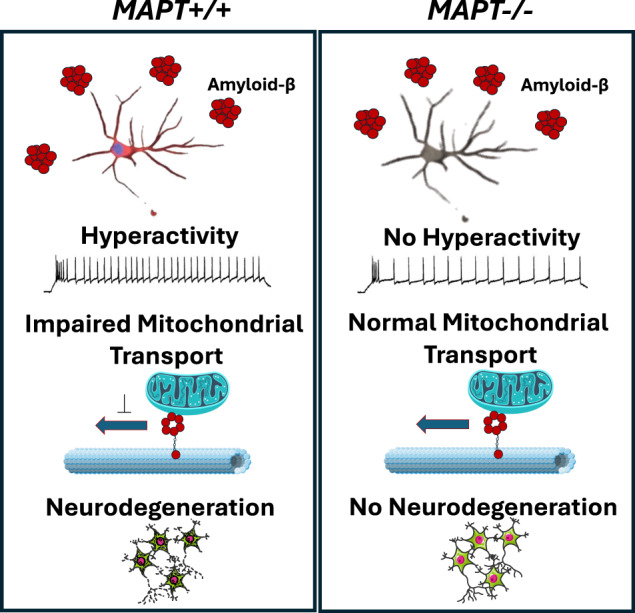

## Introduction

Alzheimer’s disease (AD) is the most common age-related neurodegenerative disease and cause of dementia, and is characterised by pathological accumulations of two key proteins – amyloid-β (Aβ) and tau – in the brain [1]. Accumulation of tau and Aβ remains the key pathology specific to AD as set out by the latest biological definition of AD in research from a National Institute on Aging-Alzheimer’s Association working group [2], although recent advances in research have identified additional systems, cell types and molecular changes that are involved in AD pathogenesis. However, the precise link between Aβ, tau and eventual neurodegeneration remains only partially understood.

Over the past few decades, researchers have shown in longitudinal studies that Aβ accumulation precedes tau pathology in AD brains and that AD brain-derived soluble Aβ induces tau hyperphosphorylation which precedes tau aggregation [3, 4]. Furthermore, disease-causing mutations in familial AD are associated with proteins involved in the pathway of Aβ production i.e., amyloid precursor protein, presenilin 1 and presenilin 2, leading to the “amyloid cascade hypothesis” [5, 6]. To investigate how tau fits into this hypothesis, mouse models with a targeted knock-out of the microtubule-associated protein tau *(Mapt)* gene have been used to demonstrate that Aβ-driven toxicity is tau-dependent as reported in studies examining neuronal activity, axonal transport, and neurodegeneration, amongst others [7–10]. *Mapt−/−* mice are viable and do not present overt cognitive or behavioural deficits, although certain strains were reported to suffer from neurite outgrowth impairment and age-dependent motor dysfunction [10–12].

Even though mouse models have been integral in elucidating mechanisms of AD pathogenesis, distinct biological differences remain between human and mouse in the context of AD. Aged mice do not develop AD pathology without the introduction of disease-causing mutations indicating a different disease susceptibility as compared to humans [13]. Even with the introduction of mutations found in familial AD cases, AD mouse models do not present with tau pathology as would be the case for typical AD pathological manifestation. One important difference between human and mouse biology is that only four-repeat tau isoforms, representing approximately half of all tau isoforms in a human brain, are expressed in adult mice [14]. It is therefore imperative to assess the role of tau in the context of Aβ-driven toxicity in a human neuronal model in addition to studies involving mouse models. However, human *MAPT−/−* cell lines have not been studied in detail to better understand tau-dependent AD pathophysiology in a human genetic background.

Induced pluripotent stem cells (iPSCs) have emerged as a versatile human cell model which can be differentiated in vitro into almost any cell type, including neurons which are normally inaccessible without invasive surgeries [15]. iPSCs can be genetically modified to represent a genotype of interest in vitro. Here, we generated stable *MAPT−/−* iPSC lines from two healthy individuals by clustered regularly interspaced short palindromic repeats (CRISPR)-Cas9-mediated gene editing. These iPSC lines were differentiated into cortical neurons and were examined for the effects of tau depletion in phenotypes that included neuronal activity, synapse loss, axonal transport, neurite outgrowth and neurodegeneration. We found that tau depletion caused significant reduction in neuronal activity but protected iPSC-derived cortical neurons from Aβ-driven neuronal hyperactivity, axonal transport deficits, and neurodegeneration.

## Methods and materials

All reagents were purchased from Sigma-Aldrich (Merck), and all cell cultures were maintained at 37 °C with 5% CO_2_ in a humidified incubator unless stated otherwise.

### Generation of *MAPT−/−* iPSC lines using CRISPR-Cas9

Two strategies targeting either Exon 1 or Exon 4 of the *MAPT* gene were employed in two separate previously published iPSC lines derived from dermal fibroblasts of healthy individuals: SBAd-03-01 (Exon 1 targeted; 31 years old female [16]; *APOE ε2/ε3*); and SFC856-03-04 (Exon 4 targeted; 76 years old female [17]; *APOE ε3/ε4*). The genetic manipulations of *MAPT* gene were achieved by using an Alt-R CRISPR-Cas9 System (Integrated DNA Technologies) following manufacturer’s protocol. The Cas9 Nuclease V3 was used with a single gRNA to result in a double-stranded break in Exon 1, whereas Exon 4 was targeted with a pair of gRNAs to create a 25 bp excision (Supplementary Table 1). To deliver hybridised Cas9-gRNA ribonucleoprotein into the iPSCs, a Neon Transfection System (Thermo) was used to transfect a suspension of 220,000 iPSCs with a single pulse at 1400 V with 20 ms pulse width. The transfected iPSCs were then plated onto one well of a 24-well plate for recovery until the cells became confluent. Thereafter, the cells were singularised before plating 2,000 cells per well in a 6-well plate on irradiated mouse embryonic fibroblast feeder cell line CF1. Ninety-six individual iPSC colonies were subsequently picked and transferred separately into a 96-well plate before they achieved confluence and were lysed for DNA extraction.

### High-content confocal microscopy imaging

#### Punctate synaptic markers

Neurons seeded in 96-well plates were imaged on a Perkin Elmer Opera Phenix high-content imager. Fifteen images were captured per well with a 43X objective at +1 µm focus level with a binning value of 1. The images were analysed with the Harmony software v4.9 from Perkin Elmer with a customised pipeline. To elaborate, the MAP2-positive neurites were identified with a 0.5-unit overall threshold as the region of interest and resized by expanding outwards by 5 px to cover synaptic signals which lay slightly above the MAP2 signals. Both presynaptic (SYNAPSIN I/II) and postsynaptic (HOMER1) signals were then identified with the Method A of the “Find spots” function with threshold values of 0.17 and 0.14, respectively, with spots larger than 100 px^2^ filtered away. Finally, the synapses were ascertained by finding HOMER1 signals in the vicinity of SYNAPSIN I/II signal regions which had been resized by expanding outwards by 5 px. The absolute number of synapses was then normalised to the total MAP2-positive area to derive synaptic density which was used for all downstream analyses. Synapse loss was achieved by adding the following Aβ insults to the neurons: (1) 10 µM of Aβ_1–42_ oligomers for 24 h, (2) 12.5% (v/v) of AD brain homogenate for 5 days or (3) AD brain-derived Aβ at 200 pg/ml for 5 days.

#### Nuclear staining such as cortical markers and cleaved caspase 3 (CC3)

Fifteen images were captured at −1, 0 and +1 and µm focus levels per well with a 20X objective and binning value of 2. The images were analysed on the same Harmony software by first identifying human nuclei among the co-culture with rat astrocytes and filtering away the nuclei with circularity less than 0.6 units. The percentage of CC3+ cells was calculated by selecting the human nuclei with mean signal intensity greater than a threshold which was determined as the mean intensity across all human nuclei identified plus two standard deviations. To induce cytotoxicity and CC3 overexpression, the neurons were treated with 10 µM of Aβ_1–42_ oligomers for 5 days.

### Live neurite tracing

To accurately trace neurite with live imaging, a low titre of lentivirus expressing both hNGN2 and green fluorescent protein (GFP) was used to transduce neural progenitor cells (NPCs) at Day 25 that led to the expression of GFP in approximately 1% of the neuronal population. The hNGN2-eGFP virus was introduced in addition to the regular Ngn2 virus on Day 25 of the cortical neuron differentiation. 0.25 μL (0.025% v/v in the neuronal media) of the hNGN2-GFP virus per well in a 12-well plate per vial of NPCs thawed, and the rest of the differentiation protocol remained the same. The neurons were seeded in co-culture with rat cortical astrocytes on Day 32 at 25,000 cells/cm^2^, and the live imaging session started on Day 35 with the neurons imaged daily for five days. For neurite tracing experiments with exogenous Aβ insults, 10 µM of Aβ_1–42_ oligomers or the scrambled control were added. The whole well was imaged with a 20X objective with 1% overlap between the fields of view to ensure neurite continuity between images.

The images were analysed with the Harmony software with a customised pipeline. Both nuclei and neurites could be identified with the same GFP signal which is stronger at the nuclei. The nuclei were first identified with a “Find nuclei” function with Method B and the identified nuclei with circularity less than 0.6, length greater than 10 µm and width to length ratio greater than 0.3 were filtered away to remove false positives. The neurites were then traced from the nuclei with a “Find neurite” function. Four parameters were selected in this study: (1) Total neurite length per neuron, (2) maximum neurite length per neuron, (3) ramification index per neuron and (4) neurite branch length per branch.

### Cytotoxicity assay (Adenylate kinase)

A ToxiLight^TM^ Non-Destructive Cytotoxicity BioAssay Kit (Lonza) was used to quantify levels of cytotoxicity in neuronal culture supernatant by measuring the release of adenylate kinase (AK). The AK detection reagent was made by mixing the lyophilised reagent with the AK assay buffer supplied in the kit at room temperature. The AK detection reagent was left for 15 min at room temperature shielded from light before 100 µL of the reagent was added to 25 µL of cell supernatant per well for 5 min at room temperature. The plate was then read in a PHERAstar^®^ microplate reader (BMG Labtech) by detecting the levels of fluorescence intensity. Higher fluorescence intensity indicates greater amount of AK released into the neuronal culture supernatant i.e. higher cytotoxicity levels. The AK assay was not performed for the experiments that involved brain homogenate treatments as the soluble factors interfered with the AK detection reagent.

### Multi-electrode array (MEA) assay

The MEA system from Axion Biosystems was used in this study. The iPSC-derived cortical neurons were seeded in the CytoView 48-well MEA plates (16 electrodes per well) on Day 32 as a droplet in the middle of a well to prevent the neurons from coming in contact with the grounding electrodes. The plates were pre-coated in poly-L-ornithine and laminin (PO/L) in a droplet and subsequently 15,000 neurons were seeded per well in a 5 µL droplet. The neurons were allowed to settle for at least 30 min before 5,000 rat astrocytes in 3 µL were added directly into the existing droplet on the plate. Finally, all cells were allowed to settle for at least 1 h before the wells were filled with 200 µL of neuronal medium on the same day. Around Day 90, baseline neuronal firing activities were first measured in the iPSC-derived cortical neurons in a Maestro Pro MEA equipment at 37 °C and 5% CO_2_ using the AxIS Navigator^TM^ v2.0.4 by recording for 6 min. The neurons were then treated with the AD brain homogenate at 25% (v/v). Neuronal activities were measured afterwards for 2 min at 6 h, 24 h, 72 h and 120 h post-treatment. The MEA data were analysed in the AxIS Navigator^TM^ and Neuro Metric Tool v2.5.1 software. All recordings were normalised to the baseline of individual well of neurons (e.g., well A1 on Day 1 to 5 of recording was normalised to the recording of well A1 at baseline).

### Live imaging of axonal transport of mitochondria

AXIS^TM^ (Millipore, discontinued in 2018) and XONA microfluidic devices were used to isolate axons and establish directionality for live imaging. Both types of devices had multiple 450-µm long microgrooves between two chambers and the cell bodies were separated by the microgrooves within each chamber. iPSC-derived cortical neurons were seeded at 100,000 cells/cm^2^ according in co-culture with confluent rat astrocytes on one side (soma chamber) of the microfluidic device. The other side of the device (distal chamber) was filled with the same neuronal media (maintained a volume gradient with less volume on the distal side) except that the concentrations for BDNF and NT-3 were ten times higher to encourage axonal outgrowth towards the distal chambers. To label mitochondria, a MitoTracker^TM^ Deep Red FM (Thermo) dye was used at 100 nM in neuronal medium for 3 h at 37 °C. The dye was removed by replacing the staining medium with Hank’s balanced salt solution supplemented with 5 mM glucose and 10 mM HEPES. A NIKON Eclipse TE-2000-U fluorescent microscope with a 20X air objective was used for live imaging by taking time lapses that lasted 150 s to capture at least twenty axons per microfluidic device. The automated microscope manoeuvre and imaging parameters were controlled by the Volocity^®^ v6.3.1 (Perkin Elmer) software.

The time course images were then exported as .tiff files to be analysed with Fiji [18] using a TrackMate macro [19] to track motile mitochondria along axons. The Laplacian of Gaussian filter was applied for the spot detector, and the estimated blob diameter was set to be 6 px. The quality threshold was selected to be 5 units and sub-pixel localisation was applied. The number of stationary spots were determined by the median value of all images and the moving spots were determined by subtracting every image in the time course to the median image. A motile mitochondrion was defined by having travelled continuously for at least 10 s and for more than 2 µm in displacement over 150 s of imaging. Motile mitochondria ratio, mean speed, and displacement of mitochondrial axonal transport were selected as readouts of interest.

For the Aβ insult experiments, baseline images were first captured post-staining before treating the neurons with 2 µM Aβ_1–42_ oligomers for 1 h at 37 °C and then imaging again post-treatment. For the positive control of axonal transport inhibition, 10 µg/ml of nocodazole was applied instead for 1 h at 37 °C.

### Mitochondrial membrane potential assay

The iPSC-derived neurons seeded at 50,000 cells/cm^2^ in monoculture were aged until Day 50. The neurons were then stained with a JC-10 dye (AAT Bioquest) before having their mitochondrial membrane potential measured by detecting signals in two wavelengths emitted by the dye. For the Aβ insult experiments, the neurons were treated with 2 µM of Aβ_1–42_ oligomers for 1 h at 37 °C before removing the oligomers and replaced the medium with DMEM basal medium without phenol red (Thermo) with JC-10 added at 1:1,000 dilution. The plate incubated at 37 °C for 1 h while shielded from light, before the dye was removed by aspiration and the neuronal culture was replenished with fresh dye-free medium.

To quantify mitochondrial membrane potential, the JC-10-treated plates were read in a PHERAstar^®^ microplate reader (BMG Labtech) by detecting the ratios of fluorescence intensity at wavelengths 520 and 590 nm. Higher 590/520 ratios indicate greater mitochondrial membrane potential with more JC-10 in aggregated form within mitochondria as compared to the monomeric form outside of mitochondria. The plate was read at baseline for three cycles each 13 min apart before adding 10 µM of carbonyl cyanide m-chlorophenyl hydrazone (CCCP) to completely depolarise mitochondrial membrane and the plate was read for another two cycles. The change in mitochondrial membrane potential (Δψ_m_) is defined as the difference in 590/520 fluorescence ratio between the baseline and depolarised mitochondria caused by CCCP.

### Statistical analyses

All data graphing and statistical tests were performed by the GraphPad Prism v9.2.0 software. NS stands for “not significant”. *p < 0.05, **p < 0.01, ***p < 0.001, ****p < 0.0001 for all statistical analyses. All data were represented as mean ± SEM. For comparisons between two groups, two-tailed Mann-Whitney test was used; for multiple groups, Kruskal–Wallis test was used to compare the groups to the *MAPT*+*/+* data with Dunn’s multiple comparison correction applied; for multiple dependent variables (for e.g. genotypes and types of treatment), two-way ANOVA was used either compared to the *MAPT*+*/+* data or the control treatment data within each *MAPT* genotype with Šídák’s (control vs single treatment/time point) or Dunnett’s (control vs multiple treatments/time points) multiple comparison correction applied. Outlier removal specifically for the axonal transport of mitochondria data was conducted with a ROUT method with 5% false discovery rate applied.

## Results

### Generation of the isogenic *MAPT−/−* iPSC panels

**Fig. 1:**
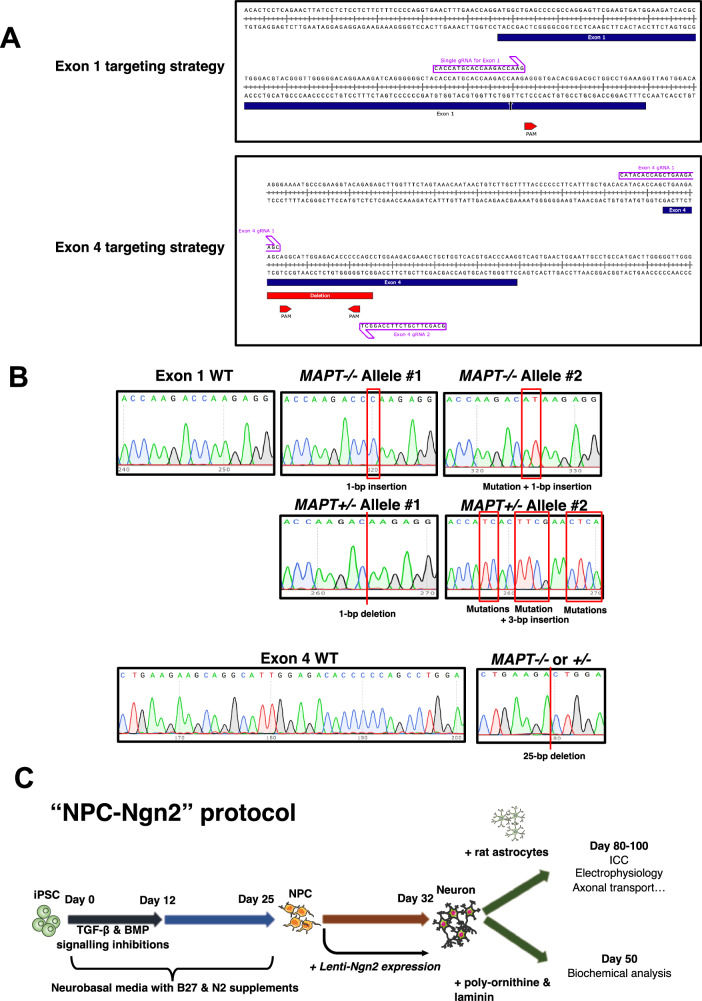
Generation of *MAPT−/−* iPSCs. **A** Illustrations of gene editing strategies showing the positions of gRNA(s) in Exon 1 and 4 DNA loci. The intended double-stranded break in Exon 1 is indicated by an arrow, whereas the intended 25-bp deletion is indicated between the gRNA pair used to target Exon 4. **B** Sequencing results of edited *MAPT* loci, including all combinations of alleles in the Exon 1 isogenic panel. For the Exon 4 isogenic panel, both *MAPT*+*/−* and *MAPT−/−* lines harbour the 25-bp deletion at the same site. **C** Schematic of the cortical neuron differentiation protocol used throughout this study – iPSC lines were first differentiated concurrently to NPCs, before they were subject to lentiviral transduction for *Ngn2* expression (“NPC-Ngn2 protocol”) for maturation either in co-culture with primary rat cortical astrocytes or in monoculture on coated surface.

To generate *MAPT−/−* iPSC lines, two parental iPSC lines from healthy individuals were used with two different targeting strategies as described in *Methods* – either a single gRNA targeting *MAPT* Exon 1 in one of the parental iPSC lines, or a pair of gRNAs targeting Exon 4 in the other, to diversify targeting options for achieving successful homozygous CRISPR-Cas9-mediated *MAPT* knockout. The intended edit was either a double-stranded break towards the end of Exon 1 3-bp upstream from the 3’-end of the gRNA binding region, or a 25-bp deletion towards the beginning of Exon 4 flanked by the pair of gRNAs used (Fig. 1A and Supplementary Table 1). After successive rounds of sequencing validations, we identified two *MAPT−/−* iPSC clones from ninety-six clones (2.1% successful targeting efficiency) of the Exon 1-targeted line and one *MAPT−/−* iPSC clone from seventy-two clones (1.4% successful targeting efficiency) of the Exon 4-targeted line. We also identified *MAPT*+*/−* (heterozygous knockout) and *MAPT*+*/+* (clones that failed to have their *MAPT* genetically edited and remained as wild-type) from each parental line to be included in downstream experiments. These isogenic iPSC lines with a range of *MAPT* genotypes are herein referred to as Exon 1 or Exon 4 isogenic panels.

The Exon 1-targeting strategy produced a range of genetic alterations due to a single DNA double-stranded break. In one of the DNA sequencing-confirmed Exon 1 *MAPT−/−* lines (*MAPT−/−* #1), one allele carries a 1-bp insertion whereas the other allele has a single-nucleotide mutation in addition to the insertion. Both genetic alterations resulted in a single-nucleotide frame shift which gave rise to a stop codon within Exon 1 (Fig. 1B; *MAPT−/−* Alleles #1 and #2). The other Exon 1 *MAPT−/−* line (*MAPT−/−* #2) has a single-nucleotide insertion as in *MAPT−/−* Allele #1 in both alleles. In the Exon 1 *MAPT*+*/−* line, one of the alleles sustained a 1-bp deletion (*MAPT*+*/−* Allele #1), similarly giving rise to a frame shift and a stop codon within Exon 1. The other allele experienced a 3-bp in-frame insertion, coupled with several mutations around the insertion site to causing changes in five amino acid residues without producing a stop codon (*MAPT*+*/−* Allele #2). The Exon 4 *MAPT−/−* and *MAPT*+*/−* lines both carry the same expected 25-bp deletion that led to a frame shift plus a stop codon within Exon 4 (Fig. 1B). A Human OmniExpress v1.2 BeadChip array (Illumina) was used post-editing to check for any gross karyotype abnormalities, and none was detected in all iPSC lines used in this study (Supplementary Fig. 1). We subsequently differentiated the Exon 1 and 4 isogenic panels into Day 30–35 NPCs and Day 50 cortical neurons as described in Supplementary Methods and illustrated in Fig. 1C, before characterising tau depletion in these isogenic panels.

### Validation of the isogenic *MAPT−/−* iPSC panels

**Fig. 2:**
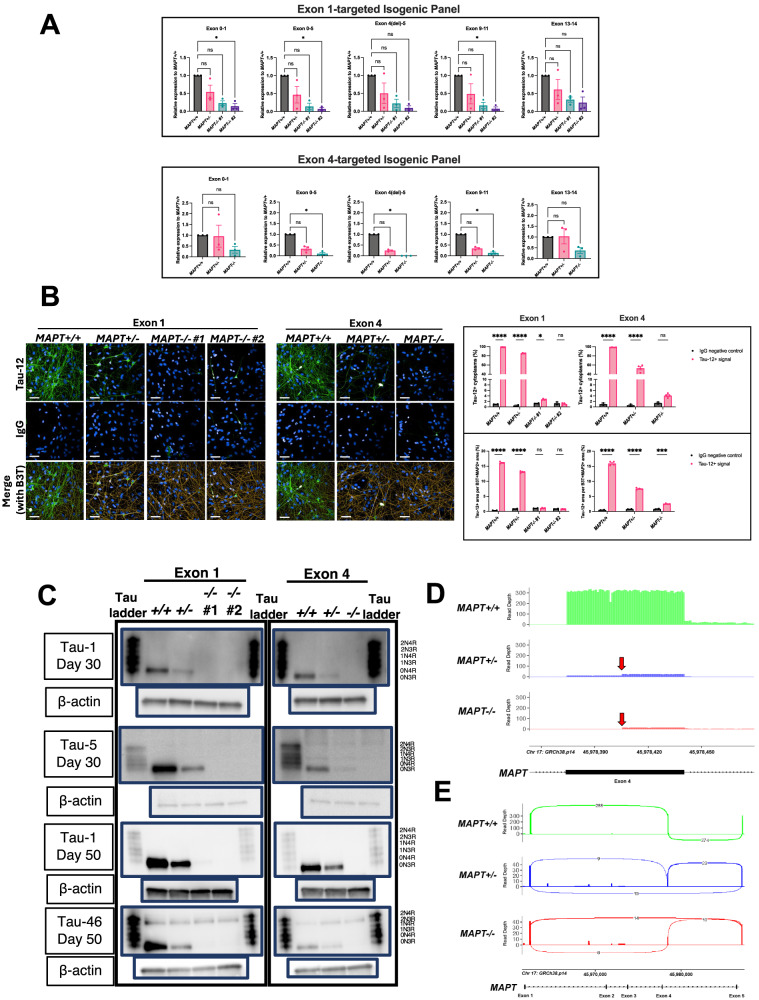
Validation of *MAPT−/−* iPSCs. **A** qRT-PCR results using cDNA samples from Day 35 NPCs of both isogenic panels. Each bar graph title indicates the primer pairs used for *MAPT* transcript detection. Exon 4(del) describes the the CRISPR targeted and excised locus. The data points were normalised to the respective *MAPT*+*/+* NPCs for each differentiation. Mean ± SEM and *n* = three independent cortical neuron differentiation repeats. Kruskal–Wallis with Dunn’s multiple comparison test was used for statistical analysis. **B** ICC of Day 50 iPSC-derived cortical neurons from both isogenic panels using Tau-12 antibody targeting to probe for total tau, showing representative images (top) and quantifications (bottom). The parameters are Tau-12+ cytoplasm normalised to the total number of nuclei (top row) and Tau-12+ area relative to the total beta-3 tubulin (B3T)+ and MAP2+ areas (bottom row). Scale bar = 50 μm. Mean ± SEM. *N* = two wells of neurons for the IgG control and four wells of neurons for the positive Tau-12 staining from one differentiation. Two-way ANOVA with Sidak’s multiple comparison test was performed for statistical analysis. **C** Western blots probing for tau using three different antibodies (Tau-1 – mid-region, Tau-5 – mid-region and Tau-46 – C-terminus) either on Day 30 (NPC) or Day 50 (neuron) of neuronal differentiation for both *MAPT−/−* isogenic panels. 6 ng of recombinant tau ladder was used and 5 μg of lysate was added per lane (except for the Tau-46 blot where 10 μg of lysate was added). Anti-β-actin blots were used as the expression control housekeeping protein for the lysates. Full blots are shown in Supplementary Fig. 3. **D** Nanopore long-read sequencing of *MAPT* transcripts in the Exon 4 isogenic panel Day 35 NPCs. Bar graph of *MAPT* transcript long-read sequencing depth (normalised across genotypes) focusing on Exon 4 showing lower abundance of *MAPT* transcripts in the *MAPT*+*/−* and *MAPT−/−* lines as compared to the *MAPT*+*/+* line. A truncated form of Exon 4 is included in a minority of reads in the *MAPT*+*/*− and *MAPT−/−* lines indicated by the red arrows. **E** Sashimi plot illustrating Exon 4 inclusion in the *MAPT*+*/+* line and skipping in the *MAPT*+*/*− and *MAPT−/−* lines. Splicing patterns supported by fewer than 10% of total reads per sample were filtered for clarity.

We collected cell lysates both for quantitative reverse-transcription PCR (qRT-PCR; Supplementary Methods and Supplementary Table 2) and western blots (Supplementary Methods and Supplementary Table 3) to probe for *MAPT* transcripts and tau proteins, respectively, in addition to fixing iPSC-derived cortical neurons for immunocytochemistry (ICC). We conducted qRT-PCR on cDNA derived from Day 35 iPSC-derived NPCs across multiple exons spanning the entire *MAPT* transcript to determine the pattern of *MAPT* expression in the *MAPT−/−* lines (Fig. 2A). Overall, a *MAPT* dose-dependent expression pattern was observed in both Exon 1 and Exon 4 isogenic panels, with marked reductions in transcript levels in the *MAPT−/−* NPCs, but not a complete elimination.

We then performed ICC on Day 50 iPSC-derived neurons by probing for total tau using a Tau-12 antibody (Fig. 2B) to further confirm dose-dependent tau depletion in all *MAPT−/−* neurons from both isogenic panels. For tau depletion characterisation by western blot, antibodies targeting either the mid-region (Tau-1 from amino acid 192 to 204 and Tau-5 from amino acid 218 to 225) or the C-terminus (Tau-46 from amino acid 404–441) of tau did not detect any 0N3R tau isoform which would be the only isoform readily expressed by the iPSC-derived NPCs and cortical neurons on Day 30 and 50, respectively (Fig. 2C).

A band corresponding to the 2N3R tau isoform was also noticeable in the Tau-46 blots across all lines, but the band was considered unspecific. Since tau isoform expression is developmentally regulated [14] i.e., expressing progressively from the shortest isoform in foetus to eventually including the full-length 2N4R tau isoform in adults, it is unlikely that the cells were expressing a more mature tau isoform without expressing the 0N3R isoform first. Furthermore, Tau-46 antibodies are known to present cross-reactivity with microtubule-associated protein 2 (MAP2) as commonly stated by commercial manufacturers, and recently shown biochemically using MAP2 peptides [20]. This likely explains the spurious band present in the Tau-46 blots as both tau and MAP2 are highly expressed in neurons. We have also probed our samples with an antibody against MAP2 and observed a strong signal which is present within the range of tau ladder like the spurious band, suggesting that the band is an isoform/truncated form of MAP2 (Supplementary Fig. 2). The extensive qRT-PCR experiments in Fig. 2A complements this observation with consistent depletion across the whole *MAPT* transcript, and therefore tau protein, in the edited lines.

We found, however, that by extending the western blot exposure time as well as saturating the chemiluminescence signals detected for the *MAPT*+*/+* lines, a non-canonical band with a molecular weight lower than that of the 0N3R (smallest) isoform became detectable in both the *MAPT*+*/−* and *MAPT−/−* lines from the Exon 4 panel especially in the relatively more mature Day 50 neurons (Supplementary Fig. 3). As this tau-immunoreactive band was not present in the *MAPT*+*/+* line, we reasoned that it may represent a non-canonical protein product of the *MAPT* gene that was generated in response to the out-of-frame deletion in Exon 4. Moreover, we noticed a faint band at the molecular weight corresponding to the 0N3R isoform position in the Exon 1 *MAPT−/−* #1 specifically in the Day 50 neurons (Tau-1 blot) that was undetectable in the original blot with the settings required to detect physiological levels of tau expression in the *MAPT*+*/+* neurons. We then further tested tau expression in Day 50 iPSC-derived cortical neurons from both isogenic panels by subjecting the cell lysates for immunoprecipitation-mass spectrometry (IP-MS) analysis. The IP-MS results indicated that tau peptides were indeed detectable in all *MAPT−/−* lines (Supplementary Fig. 4A) but the MS signal intensity (an estimate for quantity) was very low and significantly less than that in the *MAPT*+*/+* neurons at 1%, 0.06% and 11% for the Exon 1 *MAPT−/−* #1, Exon 1 *MAPT−/−* #2 and Exon 4 *MAPT−/−* neurons, respectively (Supplementary Fig. 4B). This IP-MS experiment was unable to identify the specific non-canonical tau peptide observed in the Exon 4 *MAPT*+*/−* and *MAPT−/−* lines, thus it is unclear if those tau peptides are functional. We therefore concluded that these *MAPT−/−* lines retain residual tau expression, but the expression levels are extremely low for the Exon 1 panel with an estimated ≤1% tau expression, an almost total depletion, whereas the Exon 4 *MAPT−/−* neurons expressed a non-canonical tau peptide level at approximately 11% relative to the *MAPT*+*/+* neurons.

The qRT-PCR and ICC results from Fig. 2A, B, respectively, also provide indications on the residual tau expression in the Exon 1 lines and non-canonical tau peptide in the Exon 4 lines. We observed that there is a subtle but statistically significant increase in Tau-12+ neurons relative to the IgG control background by ICC in the Exon 1 *MAPT−/− #1* line, but not the case in the overall Tau-12+ area across neurons, suggesting that the ≤1% tau expression detected from mass spectrometry comes from a minority of neurons (in approximately 1.5% of neurons relative to the IgG control). The Exon 4 *MAPT−/−* line displays higher overall Tau-12 signal as compared to the IgG control that is consistent with the western blot and mass spectrometry data. The qRT-PCR results demonstrate that the non-canonical peptide detected in the Exon 4 *MAPT−/−* line is not a result of an N- or C-terminus truncated transcript due to the targeted locus in Exon 4 because there is a detectable level across the entire transcript, but more likely a result of exon skipping since both N-terminal and C-terminal exons are detectable and the non-canonical peptide is only ~5 kD smaller than the 0N3R tau isoform (Supplementary Fig. 3). This is consistent with the mass spectrometry data demonstrating the presence oof both N-terminus and C-terminus peptides (Supplementary Fig. 4A).

To further elucidate the identity of the non-canonical transcript, we conducted an RT-PCR experiment using the cDNA templates from the Exon 4 isogenic panel NPCs with a forward and reverse primer spanning between Exon 0 and 11, respectively. The PCR products derived from the *MAPT*+*/+* line resulted in a single band just below the 800 bp marker, as expected (Supplementary Fig. 5). However, three separate bands were observed in the *MAPT*+*/−* line with the top band corresponding to the *MAPT*+*/+* band. The bottom band is situated just above the 700 bp marker, while the middle band is estimated to be less than 50 bp smaller than the top band.

We reasoned that the top and middle bands from the *MAPT*+*/−* line represent its two alleles (i.e. one of the *MAPT* loci has 25 bp excised from Exon 4). The bottom band, on the other hand, may represent the non-canonical transcript that is <100 bp smaller than the *MAPT*+*/+* transcript due to Exon 4 targeting. This interpretation is confirmed by the *MAPT−/−* line where only the bottom two bands are present, indicating that both the Exon 4-edited and the non-canonical transcripts are present. Moreover, the non-canonical transcript (bottom) in the *MAPT−/−* appears to exhibit a stronger signal as compared to that in the *MAPT*+*/−* line to suggest that it is indeed caused by Exon 4 targeting. The small <100 bp difference of the non-canonical transcript is consistent with the ~5 kD difference observed on the western blot to suggest that exon skipping is likely.

Lastly, we performed Nanopore long-read sequencing on the *MAPT* transcripts from the Day 35 Exon 4 isogenic panel NPCs with the goal of identifying the specific non-canonical transcript seen in the Exon 4 *MAPT*+*/−* and *MAPT−/−* lines. A primer targeting Exon 11 was used to reverse transcribe the *MAPT* transcripts from the 3’ end for sequencing (Supplementary Methods). We found that Exon 4 is included in every origin read in the Exon 4 *MAPT*+*/+* NPCs, while there is a marked reduction in Exon 4 read depth in the *MAPT*+*/−* and *MAPT−/−* NPCs (Fig. 2D). In the *MAPT*+*/−* NPCs, some reads span Exon 4 while others skip Exon 4 entirely or cover only part of Exon 4 until the 3’ end of the 25 bp deletion site before continuing to Exon 1 (Fig. 2D, E). Exons 3 and 2 are not incorporated into the *MAPT* transcripts suggesting that only 0 N tau isoforms are expressed. Among the small number of *MAPT* transcript reads detected in the *MAPT−/−* NPCs, however, none of the sequencing reads spans Exon 4 completely with most reads skipping Exon 4 while several cover part of Exon 4 before continuing to Exon 1 as in the case for the *MAPT*+*/−* line. The long-read sequencing results confirm that the non-canonical band seen in the western blots (Supplementary Fig. 3) and the peptides detected in the IP-MS experiment (Supplementary Fig. 4) are products of Exon 4 skipping due to Exon 4 targeting in the edited lines, consistent with the observation from the RT-PCR experiment (Supplementary Fig. 5).

### Tau depletion protects neurons from AD brain-derived Aβ-driven hyperactivity

**Fig. 3:**
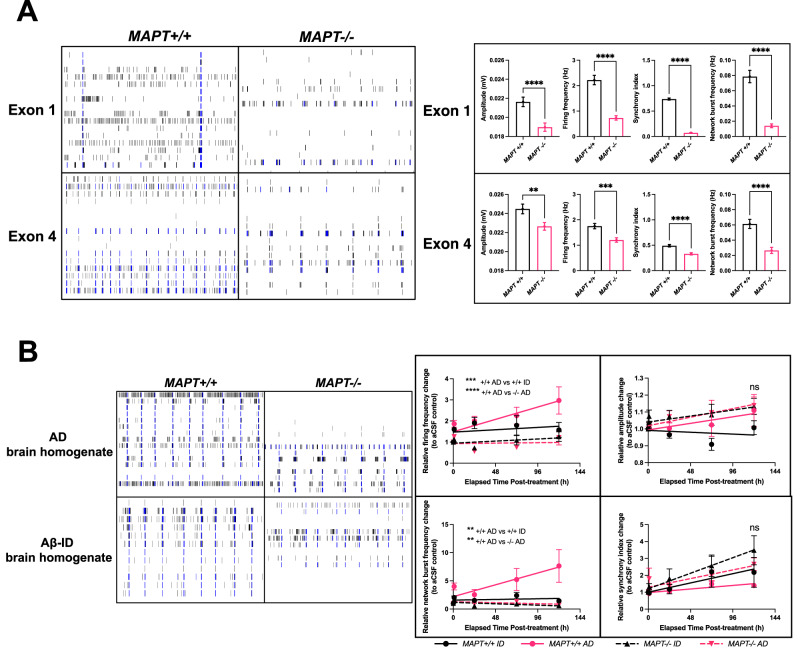
*MAPT−/−* iPSC-derived cortical neurons demonstrate reductions in neuronal activity and protection from Aβ-driven hyperactivity. **A** Representative raster plots showing individual neuronal activity spikes for each of the sixteen electrodes (row) in each MEA well over 2 min for both Day 90 *MAPT*+*/+* and *MAPT−/−* neurons (*MAPT−/−* #1 from the Exon 1 panel) from each isogenic panel at baseline; Quantification of baseline neuronal activity parameters measured by MEA assays on Day 90–100 iPSC-derived cortical neurons from both *MAPT−/−* isogenic panels. Mean ± SEM. *n* = 53–55 (Exon 1 *MAPT*+*/+*) or 51–53 (Exon 1 *MAPT−/−*) wells across three independent neuronal differentiation repeats; 101–105 (Exon 4 *MAPT*+*/+*) or 103–114 (Exon 4 *MAPT−/−*) wells across six independent neuronal differentiation repeats. Some wells did not achieve the threshold needed to register network activities. Two-tailed Mann-Whitney test was used for statistical analysis. **B** Representative raster plots showing individual neuronal activity spikes for each of the sixteen electrodes (row) in each MEA well over 2 min for both Day 90 Exon 4 *MAPT*+*/+* and *MAPT−/−* neurons treated with either AD brain homogenate or Aβ-immunodepleted (ID) AD brain homogenate at 25% v/v in the neuronal media for 5 days; Quantification of neuronal activity parameters measured by MEA assays over 5 days on Day 90–93 Exon 4 *MAPT*+*/+* and *MAPT−/−* iPSC-derived cortical neurons treated with either AD brain homogenate or Aβ-ID homogenate at 25% v/v in the neuronal media. All datapoints were normalised to the baseline recording pre-treatment, and for each time point relative to the wells subject to aCSF (vehicle) control treatment. Mean ± SEM. *n* = 7–14 (*MAPT*+*/*+ID), 11–14 (*MAPT*+*/*+AD) or 5–16 (*MAPT−/−* ID and AD) wells across three independent neuronal differentiation repeats. Two-way ANOVA with Dunnett’s multiple comparison correction was used for statistical analysis compared against the *MAPT*+*/*+AD wells 5 days post-treatment.

The Exon 1 and Exon 4 isogenic panels were then used for downstream experiments detailed in this study (Supplementary Table 4). We began by examining relatively more sensitive phenotypes such as neuronal activity and synapse loss, to axonal transport of mitochondria that involves a shorter time scale before examining relatively more severe cellular phenotypes such as neurite outgrowth impairment and neurodegeneration. To address if tau depletion affects neuronal activity, we differentiated the *MAPT*+*/+* and *MAPT−/−* lines from both Exon 1 (*MAPT−/−* #1) and Exon 4 isogenic panels into cortical neurons (Supplementary Fig. 6) on MEA multi-well plates which have sixteen electrodes embedded at the bottom of the plates per well for extracellular field potential detection (Fig. 3A). The *MAPT−/−* neurons from both Exon 1 and 4 panels exhibited marked impairments in neuronal activity across all parameters quantified i.e., showing reduced firing strength, frequency, synchronicity within the neuronal network and network firing.

We then asked whether tau lowering can protect human iPSC-derived cortical neurons from exogenous toxic insults such as Aβ by treating neurons with AD brain homogenate, or with AD brain homogenate immunodepleted for Aβ (Aβ-ID) and assaying neuronal activity by MEA (Fig. 3B). The *MAPT*+*/+* line demonstrated AD brain homogenate-driven hyperactivity over time both in terms of single-electrode neuronal firing and network firing frequencies, whereas neuronal firing amplitude and synchrony remained unaffected. This hyperactivity phenotype was not present after treatment by Aβ-ID, or after tau depletion, suggesting that the hyperactivity phenotype was specifically Aβ-driven and tau-dependent.

On the synapse level, tau depletion did not affect synaptic density in the iPSC-derived cortical neurons (Supplementary Fig. 7A and 7B). Bulk AD brain homogenate treatment led to a 20–30% synapse loss in the iPSC-derived cortical neurons regardless of their *MAPT* genotypes or the presence of Aβ (Supplementary Fig. 7C; Exon 4). We then performed extraction and concentration of Aβ from the AD brain homogenate as detailed in Supplementary Methods and showed that the treatment of AD brain-derived Aβ resulted in approximately 10% synapse loss in the *MAPT*+*/+* neurons but not in the *MAPT*+*/−* and *MAPT−/−* neurons (Supplementary Fig. 7D; Exon 4). This indicates that AD brain-derived Aβ-driven synapse loss is tau-dependent, but that there are other soluble factors present in the AD brain homogenate that can result in synapse loss. Both bulk AD brain homogenate and brain-derived Aβ treatments were insufficient to cause synapse loss in the Exon 1 isogenic panel (Supplementary Fig. 7C, D; Exon 1).

### Tau depletion mitigates Aβ-driven deficit in retrograde axonal transport of mitochondria

**Fig. 4:**
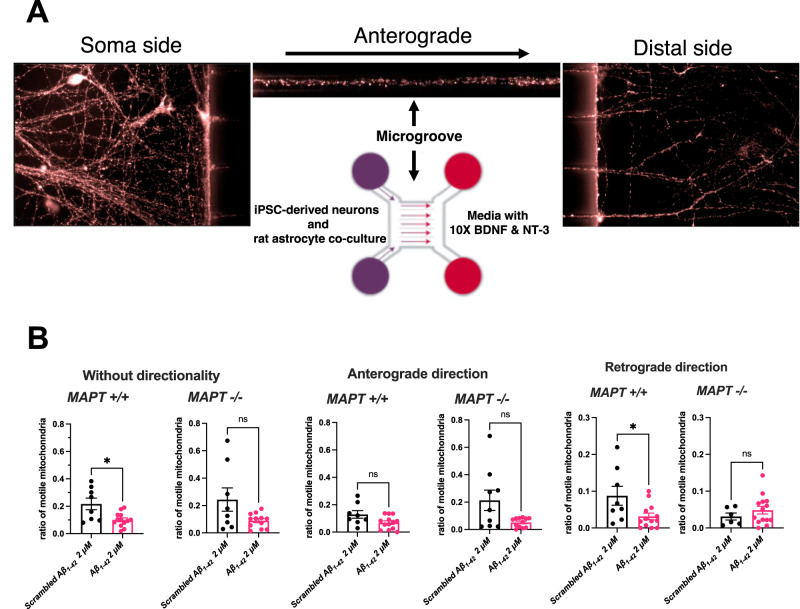
Aβ-driven retrograde impairment of axonal transport of mitochondria is absent in *MAPT−/−* iPSC-derived cortical neurons. **A** Schematic of the experiments designed to measure axonal transport of mitochondria using microfluidic chambers. **B** Quantification of ratio of motile mitochondria (motile to stationary) in Day 70–95 iPSC-derived cortical neurons from the Exon 4 isogenic panel with or without directionality over 150 s of live imaging. The neurons were treated with either 2 μM scrambled Aβ_1–42_ or Aβ_1–42_ oligomers for 1 h before imaging. Mean ± SEM. *n* = 8 (*MAPT* + */+* scrambled Aβ_1–42_), 13 (*MAPT* + */* + Aβ_1–42_), 6–9 (*MAPT−/−* scrambled Aβ_1–42_) and 12–14 (*MAPT−/−* Aβ_1–42_) microfluidic chambers measured across five independent neuronal differentiation repeats. Two-tailed Mann-Whitney test was used for statistical analysis.

Since tau is mainly localised in the axons as a microtubule-binding protein [21], we next asked whether tau depletion interferes with axonal transport of mitochondria. The iPSC-derived cortical neurons were plated in one side of microfluidic chambers for live imaging of mitochondrial movement along axons with clear directionality as detailed in *Methods* (Fig. 4A). We did not observe any changes in the ratio (to stationary mitochondria), speed and displacement of motile mitochondria in the *MAPT−/−* neurons at baseline (Supplementary Fig. 8A).

We subsequently investigated whether tau depletion mitigates neuronal response to Aβ in terms of impairments to axonal transport of mitochondria. Recombinant Aβ_1–42_ oligomers were used as a source of a more acute and toxic Aβ insult to the neurons (Supplementary Methods and Supplementary Fig. 9), as previously reported in mouse primary hippocampal neurons in vitro [8]. After one hour of exposure to Aβ_1–42_ oligomers, there were fewer motile mitochondria in the *MAPT*+*/+* neurons specifically in the retrograde direction while the Aβ-driven reduction in the number of mitochondria moving towards the soma was mitigated in the *MAPT−/−* neurons (Fig. 4B). This suggests that the neuronal response to exogenous Aβ insult in axonal transport, at least for mitochondria as a cargo, is tau dependent. The treatment of exogenous Aβ_1–42_ oligomers did not result in any changes in either speed or displacement of motile mitochondria along axons in the iPSC-derived cortical neurons regardless of their *MAPT* genotypes (Supplementary Fig. 8B). We also found that there were no differences in mitochondrial membrane potential within the Exon 1 and 4 isogenic panels at basal level, nor in response to exogenous Aβ_1–42_ oligomers, indicating that the tau-dependent phenotype in axonal transport of mitochondria observed was unrelated to mitochondrial function (Supplementary Fig. 8C, D).

### Tau depletion does not consistently result in neurite outgrowth impairments

We went on to investigate whether tau lowering can result in changes in neuronal morphology. To address this question, we transduced a subset of iPSC-derived cortical neurons with vectors expressing GFP alongside *NGN2* and tracked their individual neurite outgrowth with live imaging over time (Supplementary Fig. 10A). Both *MAPT−/−* (#1 line) neurons from the Exon 1 isogenic panel and *MAPT−/−* from the Exon 4 isogenic panel exhibited neurite outgrowth impairment with shorter neurite and axonal lengths, as well as lower ramification index (levels of branching per root from soma) as compared to the respective *MAPT*+*/+* lines without alterations in neurite branch length (Supplementary Fig. 10B, C). However, the *MAPT−/−* (#2 line) neurons from the Exon 1 isogenic panel did not suffer from any neurite outgrowth impairment and surprisingly demonstrated a reduction in neurite branch length as compared to the *MAPT*+*/+* neurons suggesting that tau depletion alone is inadequate to consistently result in neurite outgrowth impairments.

### Tau depletion protects neurons from Aβ-driven neurodegeneration

**Fig. 5:**
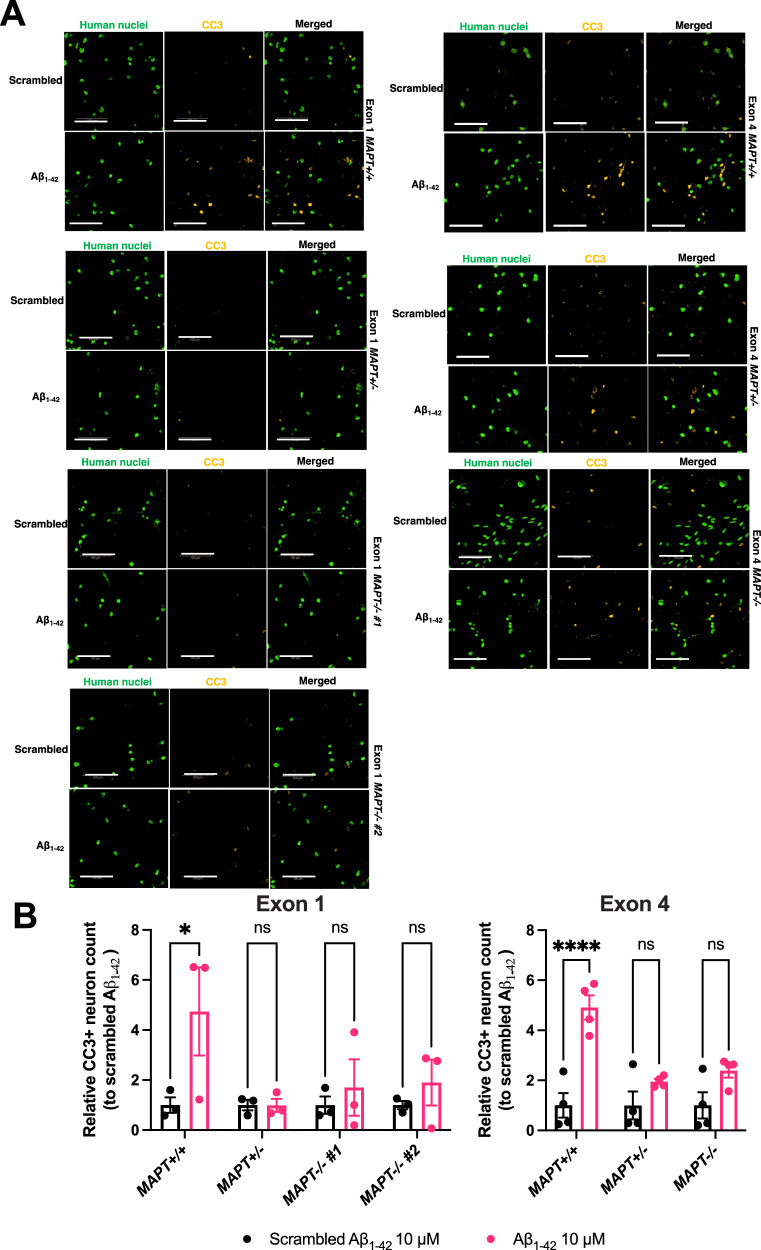
Aβ-driven neurodegeneration is absent in *MAPT−/−* iPSC-derived cortical neurons. **A** Representative immunofluorescence images of Day 79–83 (Exon 1 isogenic panel) and Day 79–86 (Exon 4 isogenic panel) iPSC-derived cortical neurons treated with either 10 μM scrambled Aβ_1–42_ or Aβ_1–42_ oligomers for 5 days. The neurons were probed with antibodies against human nuclei (green) and cleaved caspase 3 (CC3; yellow) which was used as the marker for cell death. Scale bar = 100 μm. **B** Quantification of relative CC3+ neuron count post-treatment with either 10 μM scrambled Aβ_1–42_ or Aβ_1–42_ oligomers for 5 days. Mean ± SEM. *n* = three (Exon 1) or four (Exon 4) independent neuronal differentiation repeats. Two-way ANOVA with Šídák’s multiple comparison correction was used for statistical analysis compared against the scrambled Aβ_1–42_ treatment.

Finally, we asked whether tau lowering can mitigate Aβ-driven neurodegeneration in human iPSC-derived cortical neurons. We again used recombinant Aβ_1–42_ oligomers as a more acute and toxic source of Aβ to induce neurodegeneration in the iPSC-derived cortical neurons and measured the percentage of cleaved caspase 3-positive (CC3+) neurons as a readout for cell death (Fig. 5A). In both Exon 1 and 4 isogenic panels, there was a substantial increase in Aβ_1–42_ oligomer-driven neurodegeneration in the *MAPT*+*/+* lines (Fig. 5B). This observation is supported by a different cell viability measurement using adenylate kinase (AK) (Supplementary Fig. 11). Tau depletion was effective in mitigating Aβ_1–42_ oligomer-driven neurodegeneration in both *MAPT*+*/−* and *MAPT−/−* neurons, a finding that was consistent in both isogenic panels, indicating that this phenotype is tau-dependent and, crucially, that partial tau reduction was also sufficient to mitigate Aβ_1–42_ oligomer-driven neurodegeneration.

## Discussion

In this study, we demonstrated that tau depletion in human iPSC-derived cortical neurons caused considerable reductions in neuronal activity without affecting synaptic density. We also observed neurite outgrowth impairments in two of the *MAPT−/−* lines studied. Other phenotypes, including axonal transport of mitochondria, mitochondrial function, and cortical neuron differentiation efficiency remained unaffected by tau depletion. These observations are consistent with in vivo studies conducted in *Mapt−/−* mice where the absence of tau did not lead to overt behavioural or cognitive deficiencies and mouse *Mapt−/−* primary neurons exhibited reduced baseline activity and neurite outgrowth impairment in vitro [22, 23]. Challenging iPSC-derived cortical neurons with exogenous Aβ resulted in hyperactivity, retrograde axonal transport deficit of mitochondria and neurodegeneration but, crucially, we find these adverse effects were mitigated by tau depletion.

In the presence of exogenous Aβ human iPSC-derived cortical neurons experienced hyperactivity which is tau-dependent. This is in line with clinical observations where pre-symptomatic AD patients present with hippocampal hyperactivity, as well as preclinical findings in AD mouse models where neurons in the vicinity of Aβ plaques are hyperactive, a behavioural phenotype that has been found to require tau [24–26]. It was also demonstrated recently that neuronal vulnerability to Aβ in vitro correlates with clinical vulnerability to Aβ burden manifested as hyperactivity measured by magnetoencephalography in vivo from the same AD patients [27]. Furthermore, it has been reported that *Mapt−/−* neurons were less prone to hypersynchrony caused by over-excitation and that *Mapt−/−* mice were protected from Aβ-driven seizure episodes [10, 23]. Overall, the diminished baseline neuronal activity seen in both mouse and human tau-depleted neurons may be considered a protective mechanism, rather than a functional deficit.

One of the strengths of our study is the generation and analysis of multiple *MAPT−/−* lines generated from different individuals and targeted using different strategies. Generating multiple lines allowed us to observe line-to-line variation in the response of two iPSC lines derived from different individuals to exogenous Aβ insults. Specifically, the Exon 1 isogenic panel appeared more resilient to the Aβ insults (Fig. 5B, Supplementary Fig. 7C, D) highlighting the importance of isogenic controls to eliminate from downstream experiments any effects arising from genetic variations between different individuals. Even within respective isogenic panels we still observed differences between the lines for certain phenotypes, such as neurite outgrowth impairment shown in one of the two Exon 1 *MAPT−/−* lines. This inconsistency in neurite outgrowth has also been observed in different *Mapt−/−* mouse strains and it has been suggested that compensatory expression from other microtubule-associated proteins could potentially account for the difference, although it is unclear how this is regulated in different *Mapt−/−* mouse strains [22, 28].

A challenge in any study modelling AD in vitro is the choice of exogenous Aβ insult applicable across a range of phenotypic assays. We found AD brain homogenate to be a physiological source of pathologically relevant Aβ for electrophysiological and synaptic assays with a concentration of Aβ in the pM range. AD brain homogenate had been routinely used in other electrophysiology studies and we found that the hyperactivity readout in human iPSC-derived cortical neurons was particularly sensitive to and specifically caused by the presence of Aβ. However, we did not observe such specificity in the synapse loss experiment, suggesting that other soluble factors present in the AD brain homogenate could similarly lead to synapse loss (Supplementary Fig. 7C). Treating neurons with purified AD brain-derived Aβ demonstrated that the Exon 4 *MAPT−/−* neurons were protected from Aβ-driven synapse loss, although 200 pg/ml of AD brain-derived Aβ was insufficient to cause synapse loss in the Exon 1 isogenic panel (Supplementary Fig. 7D). However, the use of AD brain-derived Aβ is limited by the capture-elution efficiency of the current method in which 1 g of AD brain tissue was needed to extract 1 ng of AD brain-derived Aβ.

Recombinant Aβ_1–42_ oligomers provide a higher concentration of exogenous Aβ resulting in more robust phenotypes. Retrograde axonal transport of mitochondria was impaired by 2 μM of Aβ_1–42_ oligomers in iPSC-derived cortical neurons, consistent with a previous study in mouse primary hippocampal neurons with a similar experimental setup [8]. However, in that previous study axonal transport was impaired in both directions and it was later reported that naturally occurring Aβ peptides in an AD mouse model led to axonal transport deficits only in the anterograde direction [29]. The consensus between the present human cell model study and previous mouse studies is that Aβ drives a reduction in the number of motile mitochondria transported along axons, although specific effects of Aβ may be dependent on the experimental setup, source of Aβ and/or species of cellular model.

To cause a robust neurodegeneration cell death phenotype, we treated iPSC-derived cortical neurons with 10 μM of Aβ_1–42_ oligomers for five days. This supraphysiological insult consistently resulted in at least two-fold increase in the percentage of CC3-positive neurons, even in the Exon 1 isogenic panel which was more resilient to Aβ challenges (Fig. 5). Although there is broad agreement when using the AK-based cell viability assay, the Exon 4 *MAPT−/−* neurons also exhibit poorer cell viability in that assay (Supplementary Fig. 11), compared to the data on CC3 staining. It is important to note that the assays are not equivalent and that AK is detected due to its release from intracellular space caused by plasma membrane rupture whereas CC3 is an intracellular marker for programmed cell death initiation. While the CC3 quantification represents cell death specifically in the iPSC-derived neurons labelled by the human nucleus antigen, AK measurements can potentially be confounded by Aβ_1–42_ oligomer-driven plasma membrane disruption [30] as well as cell death in the rat astrocyte population in the co-culture, hence masking the true readouts of neuronal viability.

Nevertheless, we observed similar patterns in both assays where tau-expressing neurons were more susceptible to Aβ as compared to tau-depleted neurons especially in the Exon 1 panel where both *MAPT−/−* neuronal cultures do not show Aβ-driven loss of cell viability measured by the release of AK. The *MAPT*+*/−* and *MAPT−/−* neurons from both isogenic panels consistently demonstrated protection from Aβ-driven neurodegeneration in line with a previous study in *Mapt−/−* primary hippocampal neurons in which 20 μM of Aβ_1–40_ fibrils was used to treat neurons for four days [9]. This critical phenotype highlights consistent protective effects of tau lowering in Aβ-driven toxicity in AD pathogenesis in human and mouse cells.

Another strength of our study was to comprehensively investigate tau expression levels in *MAPT−/−* lines with IP-MS methodology in addition to qRT-PCR, long-read sequencing, ICC, and western blots (Fig. 2 and Supplementary Figs. 3–5). We found that the Exon 1 *MAPT−/−* lines carrying single nucleotide insertions expressed extremely low levels (≤1%) of tau peptides as compared to the *MAPT*+*/+* neurons, an almost total depletion that is also corroborated by the ICC results (Fig. 2B). The Exon 4 *MAPT−/−* line (carrying a 25 bp deletion) produced a non-canonical tau-immunoreactive band of a molecular weight lower than that of the smallest 0N3R tau isoform that likely arose through skipping of Exon 4 as indicated by the qRT-PCR and RT-PCR data (Fig. 2A and Supplementary Fig. 5), an event that was previously reported and would neither introduce an early stop codon nor alter the sequence of the remainder of the tau protein [31], at ~11% of overall expression levels as compared to the isogenic *MAPT*+*/+* neurons. We subsequently confirmed with Nanopore long-read sequencing that the identity of the non-canonical transcript/peptide observed in the Exon 4 targeted lines is indeed a product of Exon 4 skipping (Fig. 2D, E). Nevertheless, marked tau depletion was achieved in the Exon 4 *MAPT−/−* lines and all phenotypes reported in the Exon 1 *MAPT−/−* lines were consistently reproduced in the Exon 4 *MAPT−/−* line, suggesting the functional effects observed were specifically caused by chronic tau depletion.

## Conclusions

In this study, we established stable human iPSC isogenic panels with chronic tau depletion from two healthy individuals. We demonstrated that a wide range of Aβ-driven phenotypes in iPSC-derived cortical neurons were tau-dependent, including Aβ-driven hyperactivity, axonal transport deficits, and neurodegeneration, consistent with studies conducted in *Mapt−/−* mouse models. Our findings highlight the potential benefits of ongoing attempts at chronic tau-lowering strategies in AD in the clinic. iPSC-derived *MAPT−/−* human cortical neurons can be applied to investigate the involvement of tau in Aβ-driven toxicity in cortical neurons and in other tauopathy-relevant pathways.

## Supplementary information

Supplementary materials

## Data Availability

The datasets used and/or analysed during the current study are available from the corresponding author on reasonable request. The iPSC lines used in this study can be requested from the James and Lilian Martin Centre for Stem Cell Research.
