# Supplementary material for: Tau depletion in human neurons mitigates Aβ-driven toxicity
Source: Mol Psychiatry. 2024 Feb 15;29(7):2009–20. doi: 10.1038/s41380-024-02463-2 (PMC11408257; doi:10.1038/s41380-024-02463-2)
Supplement: Supplementary file 1 — Supplementary materials [file 41380_2024_2463_MOESM1_ESM.pdf]

Supplementary materials for

Tau depletion in human neurons mitigates Aβ-driven toxicity

Bryan Ng, Jane Vowles, Féodora Bertherat, Ajantha Abey, Peter Kilfeather, Dayne Beccano-Kelly, M Irina Stefana, Darragh P. O'Brien, Nora Bengoa-Vergniory, Phillippa J. Carling, John A. Todd, Tara M. Caffrey, Natalie Connor-Robson, Sally A. Cowley* and Richard Wade-Martins*

* co-corresponding authors:

Richard Wade-Martins

richard.wade-martins@dpag.ox.ac.uk

Department of Physiology, Anatomy and Genetics

Kavli Institute for Nanoscience Discovery

University of Oxford

South Parks Road

Oxford OX1 3QU

United Kingdom

Sally A. Cowley

sally.cowley@path.ox.ac.uk

James and Lillian Martin Centre for Stem Cell Research

Sir William Dunn School of Pathology

University of Oxford

South Parks Road

Oxford OX1 3RE

United Kingdom

This PDF contains:

Supplementary Tables 1-4

Supplementary Figures 1-11

Supplementary methods and materials

Supplementary Table 1:

gRNA used (shown as DNA sequence) for targeting the *MAPT* gene with CRISPR-Cas9 system and sequencing primers spanning the targeted regions

gRNA target	Sequence (5' – 3')
MAPT Exon 1	CACCATGCACCAAGACCAAG
MAPT Exon 4 (forward)	CATACACCAGCTGAAGAAGC
MAPT Exon 4 (reverse)	GCAGCTTCGTCTTCCAGGCT
**Primer target**	**Sequence (5' – 3')**
MAPT exon 1 (forward)	AATATGGAGCACGGGATGAG
MAPT exon 1 (reverse)	CCGTGTCTGGCCATTATCTC
MAPT exon 4 (forward)	AGAGCTGAGGCACCTTGGTA
MAPT exon 4 (reverse)	CCCGGCCTAGAGTTTCTGTT
T7 (pGEM-T Easy vector)	TAATACGACTCACTATAGGG
SP6 (pGEM-T Easy vector)	TATTTAGGTGACACTATAG

Supplementary Table 2:

Primer sequences (5’ to 3’) used for the qRT-PCR experiments on the *MAPT* transcripts. (F): Forward primer and (R): Reverse primer.

Primer target	Forward primer	Reverse primer
GAPDH	CTGACTTCAACACGCACACC	ATGAGGTCCACCACCCTG
***MAPT* Exon 0 (F) to Exon 1 (R)**	CCTCGCCTCTGTCGACTATC	TACGTCCCAGCGTGATCTTC
***MAPT* Exon 0 (F) to Exon 5 (R)**	CCTCGCCTCTGTCGACTATC	GCTTTTACTGACCATGCGAG
***MAPT* Exon 4 (F) to Exon 5 (R)**	AGGCATTGGAGACACCCCC	CATCGCTTCCAGTCCCGT
***MAPT* Exon 9 (F) to Exon 11 (R)**	AAGAGCCGCCTGCAGACA	TGGTTTATGATGGATGTTGCCTAA
***MAPT* Exon 13 (F) to Exon 14 (R)**	GACAGGCTTTCCCAGGCAG	GGACCCCTGAAAGAGGGCAGC

Supplementary Table 3: Primary and secondary antibodies used for western blot and immunocytochemistry

Target of primary antibodies for western blot	Dilution factor	Manufacturer and catalogue number
Tau-1	1:1000	Merck MAB3420
Tau-5	1:500	Abcam ab80579
Tau-46	1:500	Santa Cruz sc-32274
MAP2	1:500	Abcam ab5543
Anti-Aβ clone 6E10	1:5,000	Biolegend #803015
Actin conjugated to HRP	1:20,000	Abcam ab49900
**Secondary antibody for western blot**	**Dilution factor**	**Manufacturer and catalogue number**
Goat anti-mouse IgG (H+L) HRP conjugate	1:5,000	Bio-Rad 1706516
**Target of primary antibodies for immunocytochemistry**	**Dilution factor**	**Manufacturer and catalogue number**
Synapsin I/II	1:500	Synaptic Systems 106004
Homer1	1:500	Synaptic Systems 160003
MAP2	1:1,000	Abcam ab92434
Human nuclear antigen	1:200	Abcam ab191181
Cleaved caspase 3	1:500	Abcam ab2302
CUX2	1:200	Abcam ab130395
TBR1	1:200	Abcam ab31940
CTIP2	1:500	Abcam ab18465
Tau-12	1:250	Merck MAB2241
**Secondary antibodies for immunocytochemistry**	**Dilution factor**	**Manufacturer and catalogue number**
Goat anti-guinea pig Dylight 488	1:1,000	Abcam ab96959
Goat anti-mouse Alexa Fluor 488	1:1,000	Thermo A11001
Goat anti-rabbit Alexa Fluor 555	1:1,000	Thermo A27039
Goat anti-chicken Alexa Fluor 555	1:1,000	Thermo A21437
Goat anti-rabbit Alexa Fluor 647	1:1,000	Thermo A21245
Goat anti-rat Alexa Fluor 647	1:1,000	Thermo A21247

Supplementary Table 4: Specific iPSC lines used for downstream experiments on iPSC-derived cortical neurons

			iPSC lines used in specific experiments:			
**iPSC clone identifier**	**Reference in text**	**CRISPR-targeted exon**	**MEA**	**MEA – AD brain homogenate treated**	**Mitochondrial transport**	**Cytotoxicity, synapse quantification, neurite tracing & mitochondrial membrane potential**
SBAd-03-01 – B3 clone	*MAPT+/+* line	Exon 1	✓			✓
SBAd-03-01 – G9 clone	*MAPT+/-* line	Exon 1				✓
SBAd-03-01 – A1 clone	*MAPT-/-* #1 line	Exon 1	✓			✓
SBAd-03-01 – F5 clone	*MAPT-/-* #2 line	Exon 1				✓
SFC856-03-04 – B7 clone	*MAPT+/+* line	Exon 4	✓	✓	✓	✓
SFC856-03-04 – A12 clone	*MAPT+/-* line	Exon 4				✓
SFC856-03-04 – E1 clone	*MAPT-/-* line	Exon 4	✓	✓	✓	✓

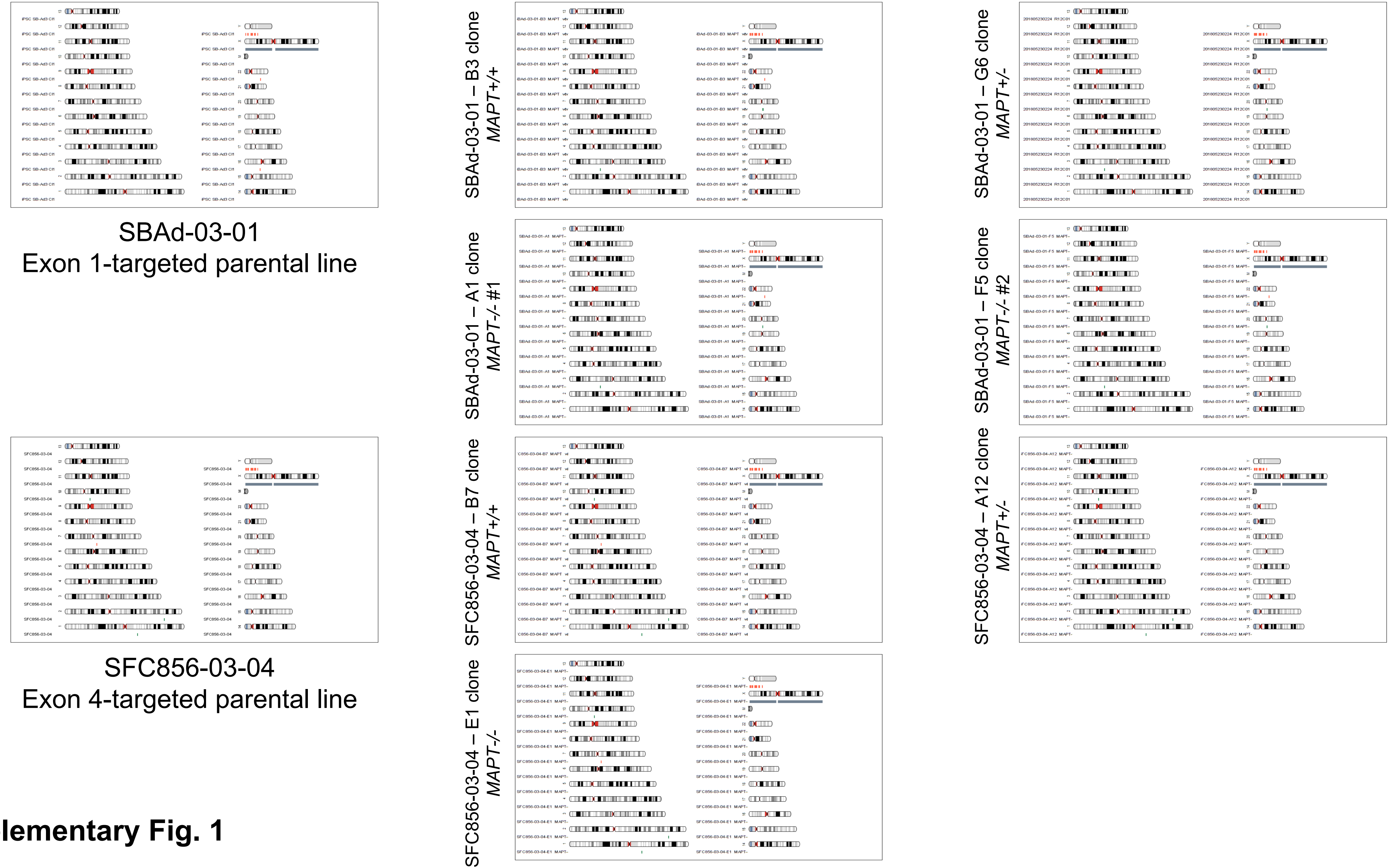

Supplementary Fig. 1

Supplementary Figure 1: Quality control of iPSC lines used in this study.

Genome integrity of the parental iPSC lines and both isogenic panels that underwent CRISPR-Cas9 gene editing targeting *MAPT*, examined by the Illumina OmniExpress24 single nucleotide polymorphism array (except the parental SBAd-03-01 line which was examined by the Illumina CytoSNP-12 array). Karyograms (KaryoStudio, Illumina) show amplifications (green)/deletions (orange)/loss of heterozygosity regions (grey) alongside the relevant chromosome. Female X chromosome is annotated in grey.

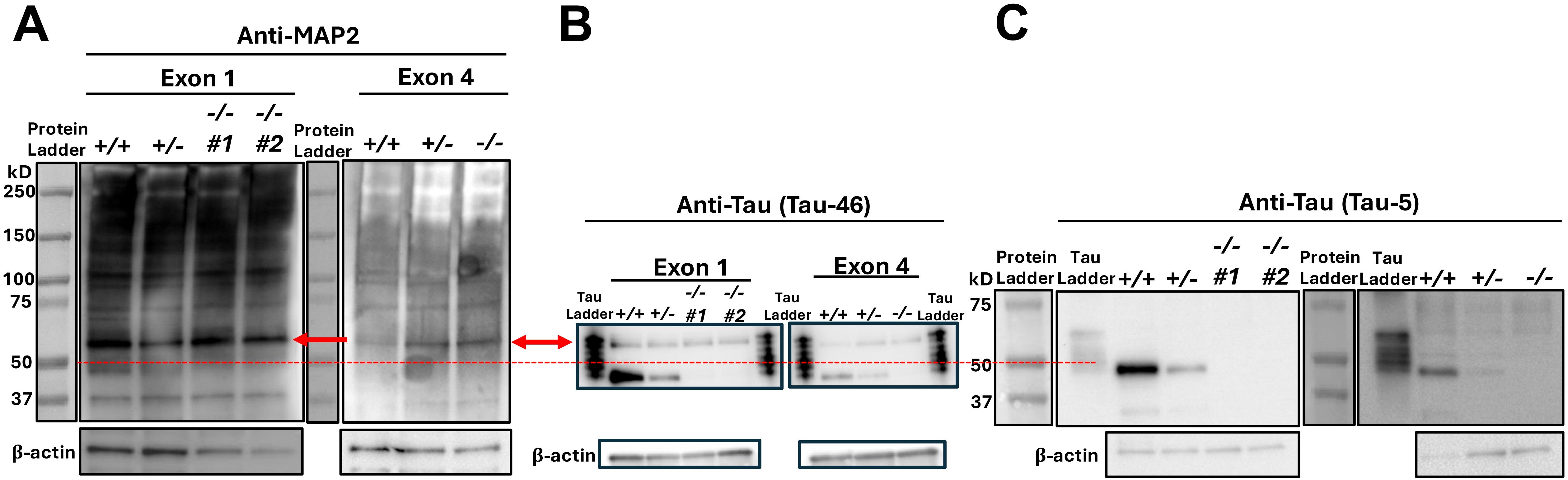

Supplementary Fig. 2

Supplementary Figure 2: Western blots probing for MAP2 and total tau in 10 μg lysate of Day 50 neurons from both isogenic panels.

The western blot probing for MAP2 **(A)** is aligned with both Tau-46 **(B)** and Tau-5 **(C)** blots extracted from Fig. 2C. Red arrows indicate the position of the MAP2 bands of interest within the range of tau ladder in alignment with the spurious bands found in the Tau-46 blot. The dotted line marks the 50 kD/tau ladder alignment across three different blots.

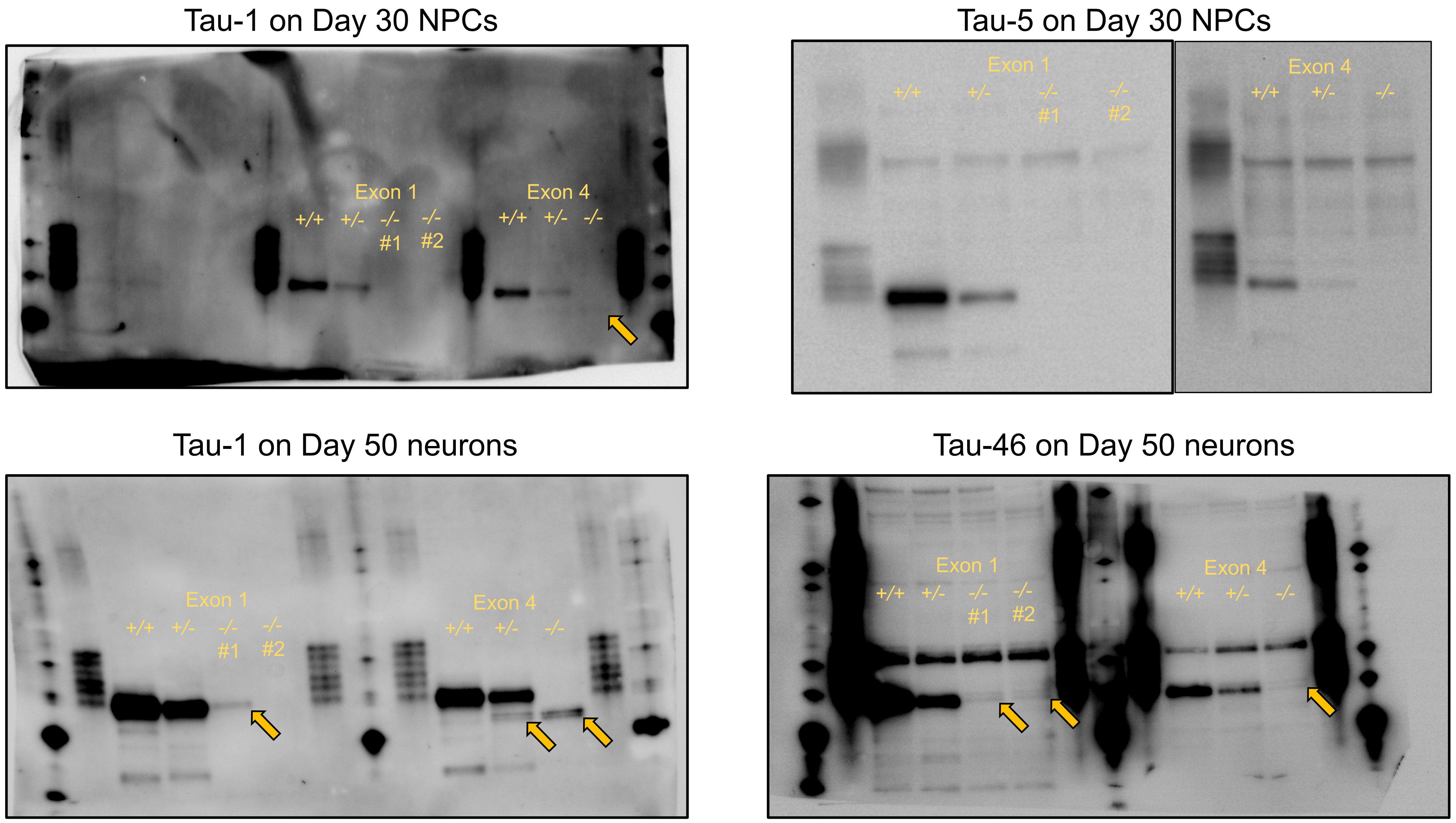

Supplementary Fig. 3

Supplementary Figure 3: Full western blots as in Fig. 2C with artificial signal augmentation.

Western blots with either Day 30 NPCs or Day 50 neurons for all seven iPSC lines used in this study probed by Tau-1 (mid-region), Tau-5 (mid-region) and Tau-46 (C-terminus) antibodies. The bands in question are indicated by yellow arrows. Recombinant tau ladders flanked the labelled lines.

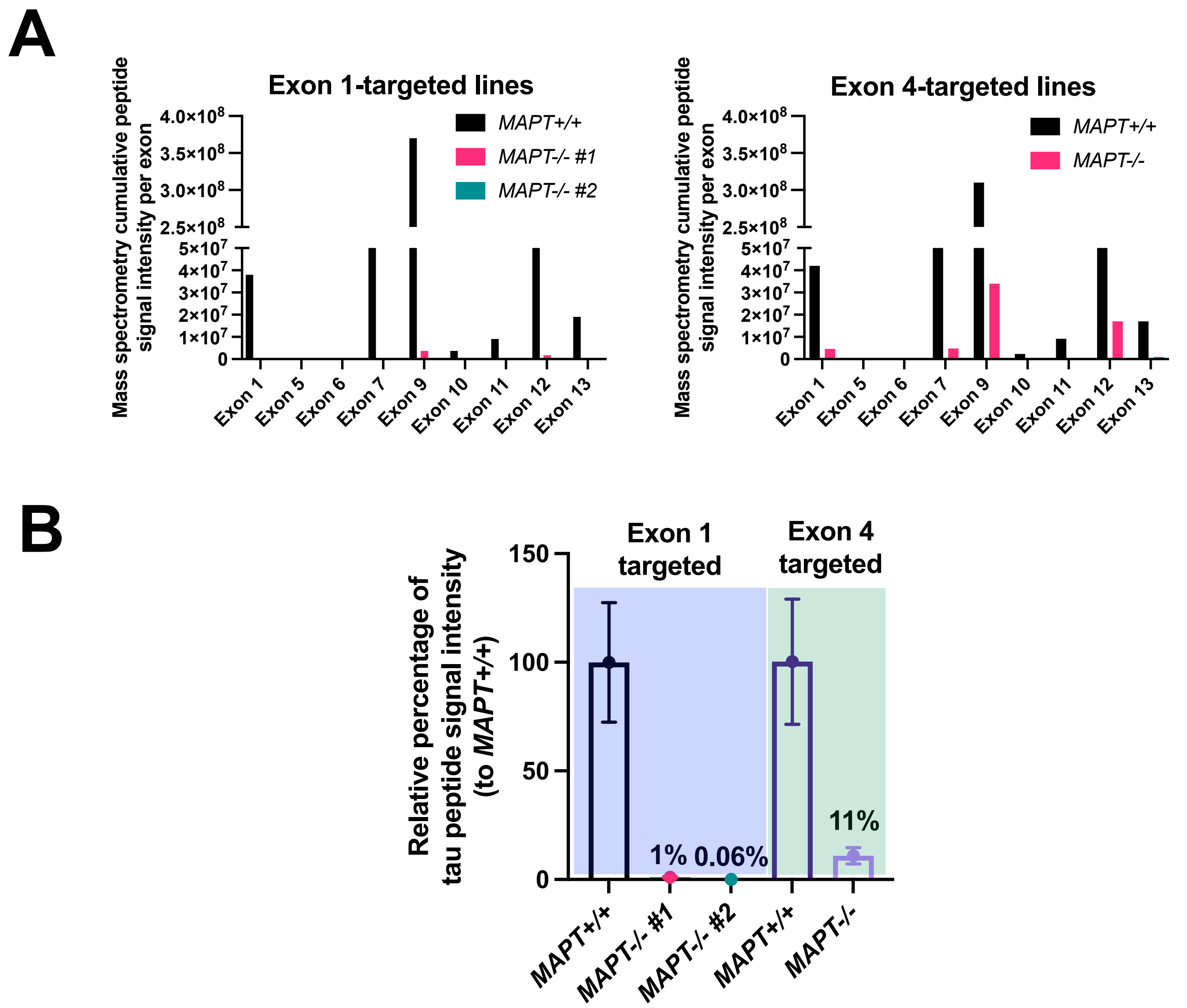

Supplementary Fig. 4

Supplementary Figure 4: IP-MS experiment identified tau peptides present in Day 50 *MAPT-/-* neurons.

(A) Quantifications of cumulative tau peptide signal intensity per exon identified from the IP using polyclonal K9JA antibody (Dako) binding to the C-terminus of tau in the *MAPT+/+* neurons compared to the *MAPT-/-* neurons.

(B) Quantifications of relative tau peptide signal intensity percentage to those in the respective *MAPT+/+* neurons. Mean ± SEM. *n* = 51 tau peptides identified in the *MAPT+/+* neurons from the IP-MS analysis.

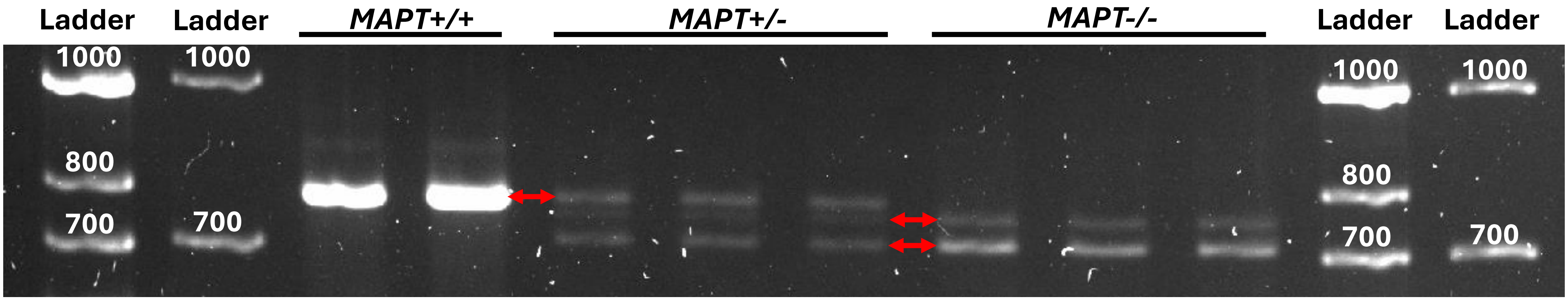

Supplementary Fig. 5

Supplementary Figure 5: RT-PCR on Day 45 NPCs from the Exon 4-targeted isogenic panel.

DNA agarose gel image of RT-PCR products using cDNA as the template using *MAPT* Exon 0 (forward) and Exon 11 (reverse) targeting primers. The red double-arrows indicate the three bands of interest.

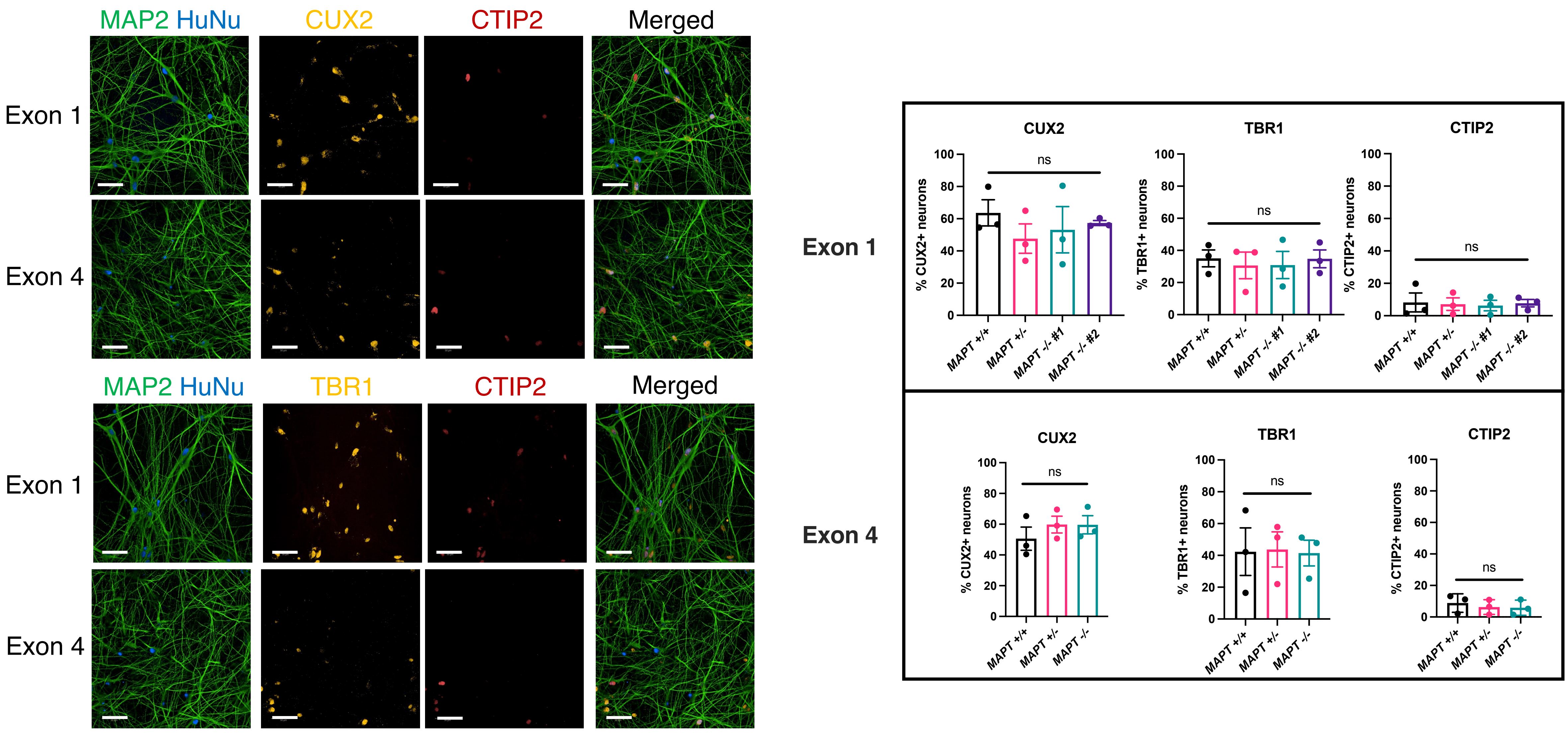

Supplementary Fig. 6

Supplementary Figure 6: Cortical marker expression levels of the iPSC-derived neurons used in this study.

Representative fluorescent images of Day 80 iPSC-derived cortical neurons from the *MAPT+/+* lines in co-culture with primary rat cortical astrocytes (Scale bar = 50 μm), as well as quantifications showing the quantification of cortical marker expression levels in Day 73-80 (Exon 1) or Day 80-84 (Exon 4) neurons. CUX2 (upper cortical layer), TBR1 (deeper cortical layer) and CTIP2 (deeper cortical layer, early marker) were used for staining in human nuclear antigen-positive cells. Mean ± SEM. *n* = three independent cortical neuron differentiation repeats. Kruskal-Wallis test with Dunn's multiple comparison correction was used for statistical analysis.

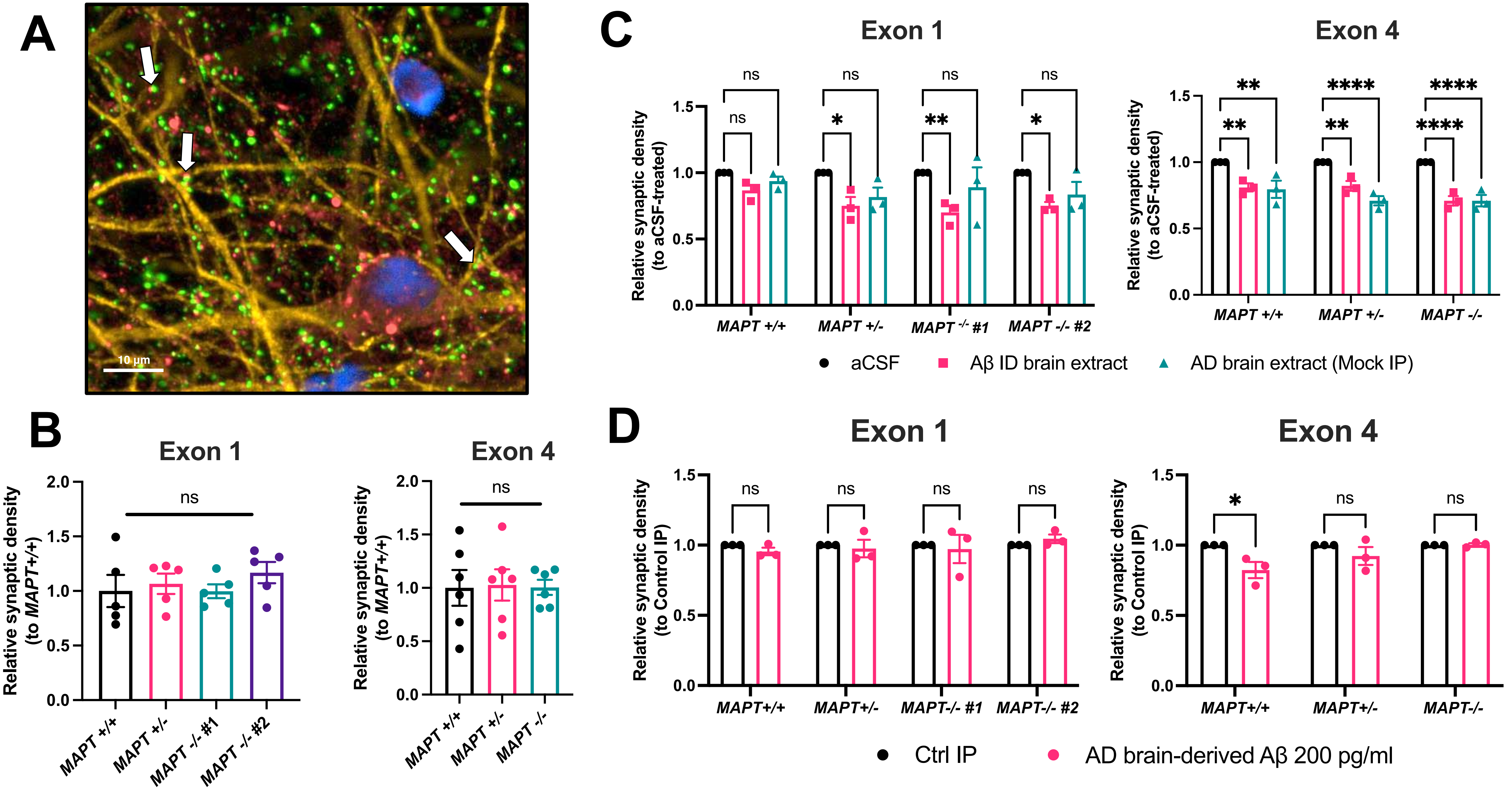

Supplementary Fig. 7

Supplementary Figure 7: Aβ-driven synapse loss in iPSC-derived cortical neurons.

(A) Representative fluorescent image of Day 88 iPSC-derived cortical neurons labelled with presynaptic Synapsin I/II (green), postsynaptic Homer1 (red), MAP2 (yellow) and human nucleus (blue). Synapsin I/II and Homer1 spots were identified in apposition within MAP2-positive regions to be considered a synapse indicated by white arrows. Scale bar = 10 μm.

(B) Quantifications of relative synaptic density in Day 74-89 iPSC-derived cortical neurons compared to the respective *MAPT+/+* line at baseline. Mean ± SEM. *n* = five (Exon 1) or six (Exon 4) independent neuronal differentiation repeats. Kruskal-Wallis test with Dunn's multiple comparison correction was used for statistical analysis.

(C) Quantifications of relative synaptic density in Day 74-89 iPSC-derived cortical neurons treated with 12.5% (v/v) AD or Aβ-ID brain homogenate for five days compared to the control aCSF treatment within each genotype. Mean ± SEM. *n* = three independent neuronal differentiation repeats. Two-way ANOVA with Dunnett's multiple comparison correction was used for statistical analysis.

(D) Quantifications of relative synaptic density in Day 79-86 iPSC-derived cortical neurons treated with either 200 pg/ml of AD brain-derived Aβ obtained from IP using antibodies targeting Aβ or equal volume of eluate from control IP using normal mouse IgG for five days. Mean ± SEM. *n* = three independent neuronal differentiation repeats. Two-way ANOVA with Šídák's multiple comparison correction was used for statistical analysis.

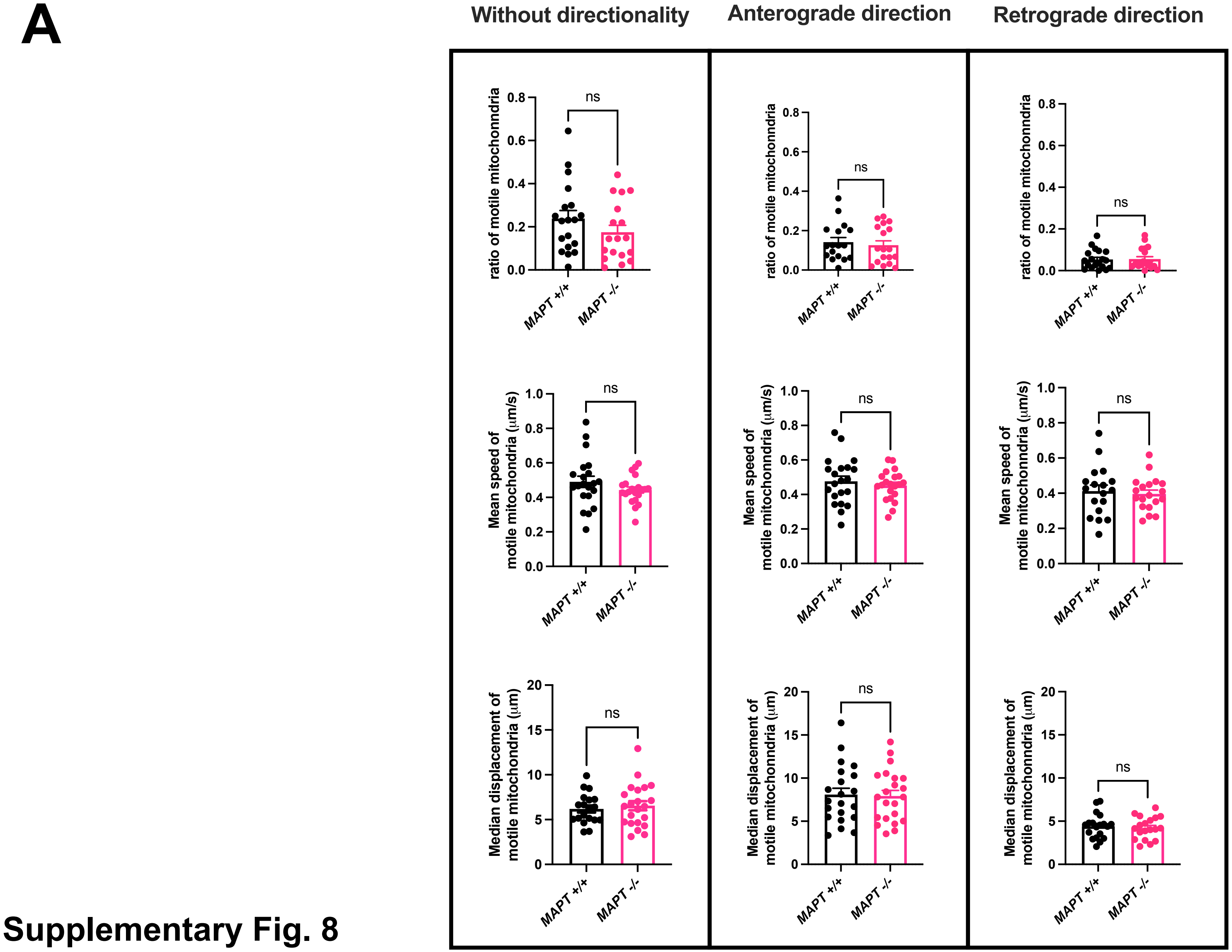

Supplementary Fig. 8

B

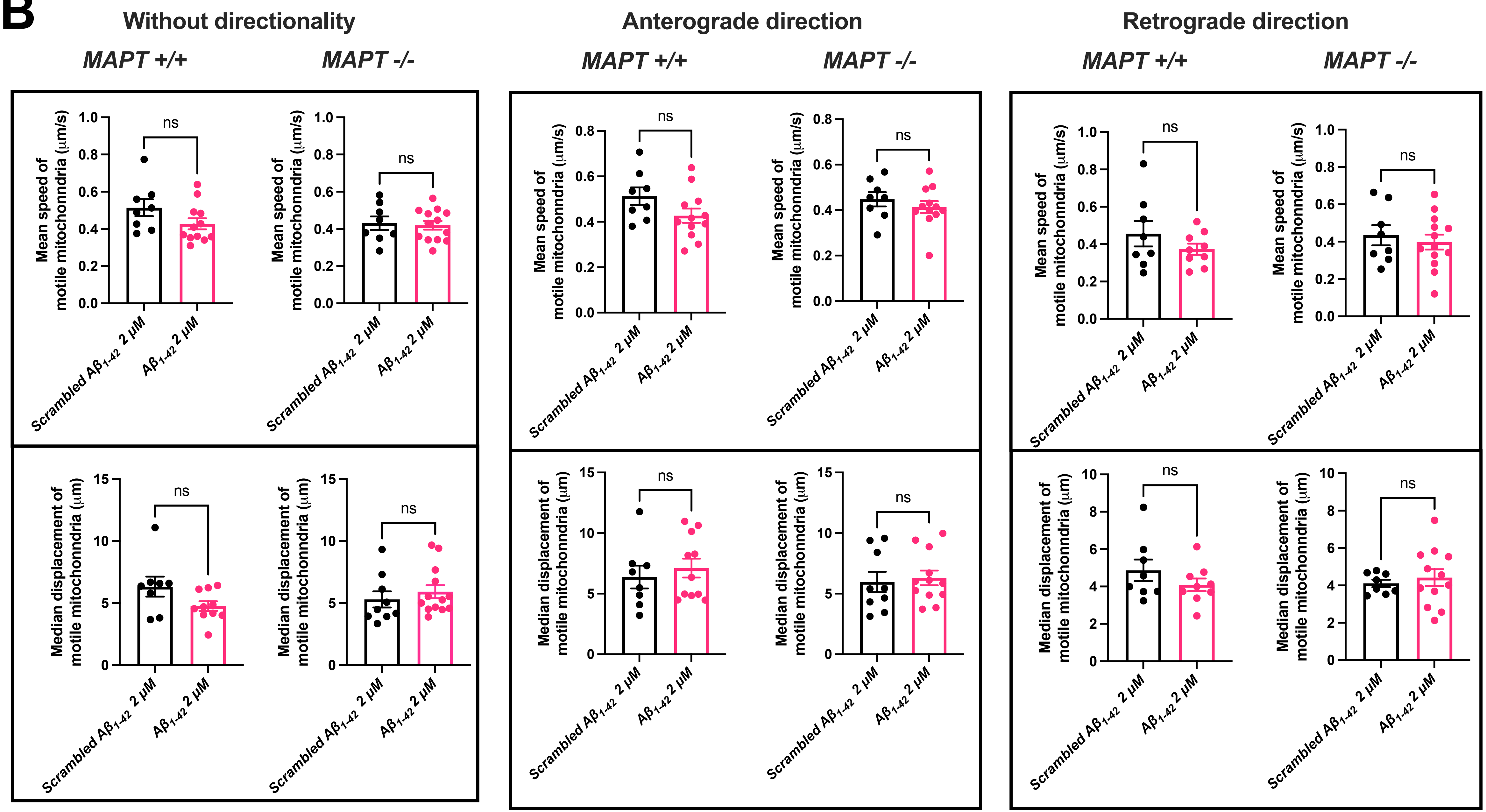

Supplementary Fig. 8

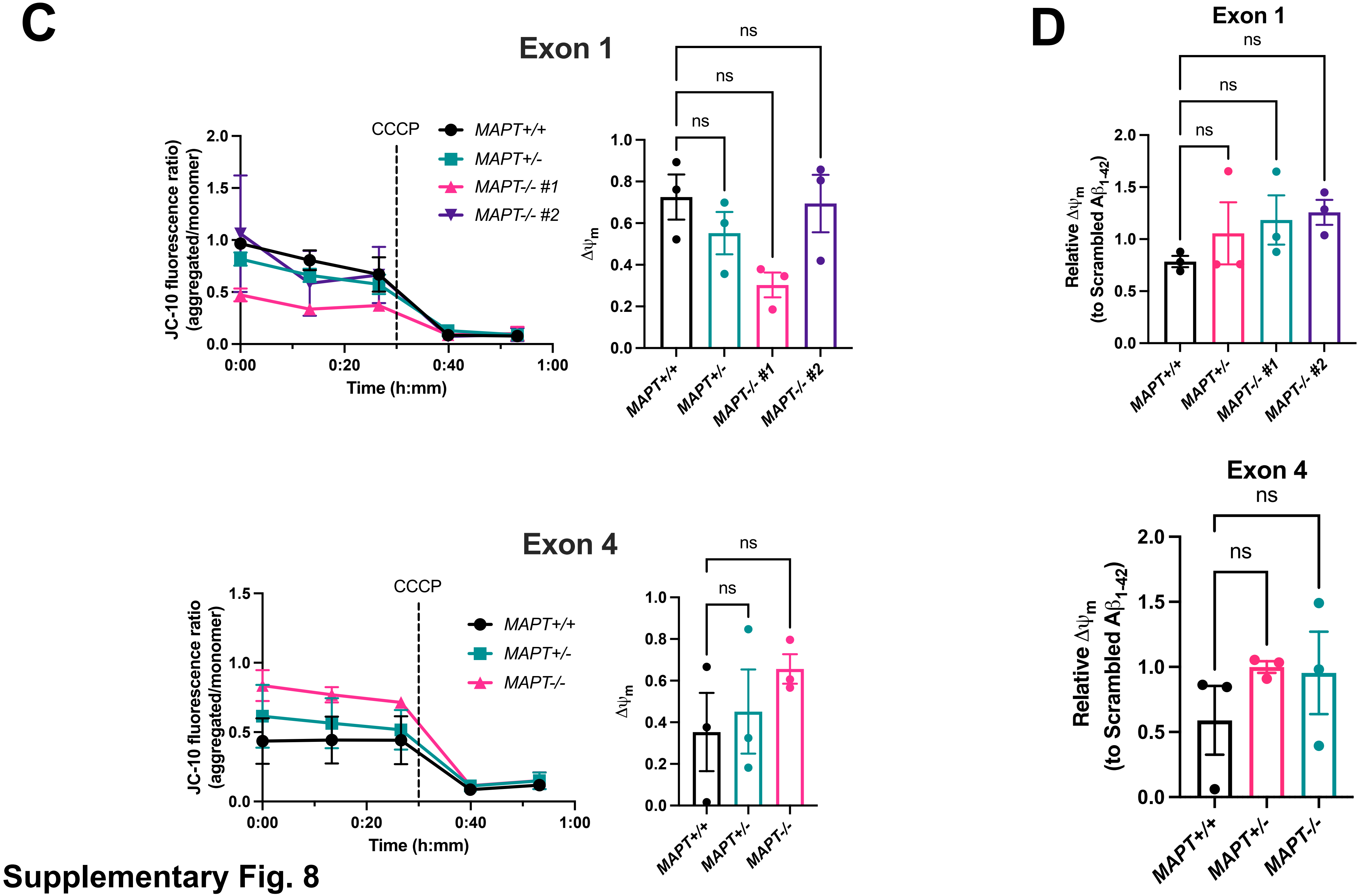

Supplementary Fig. 8

Supplementary Figure 8: Axonal transport of mitochondria and their membrane potential in iPSC-derived cortical neurons.

(A) Quantifications of ratios (to stationary mitochondria), speed and displacement of motile mitochondria at baseline with or without directionality over 150 s of live imaging in Day 70-95 iPSC-derived cortical neurons from the Exon 4 isogenic panel. Mean ± SEM. *n* = 17-21 (*MAPT+/+*) or 18-22 (*MAPT-/-*) microfluidic chambers measured across five independent neuronal differentiation repeats. Two-tailed Mann-Whitney test was used for statistical analysis.

(B) Quantifications of speed and displacement of motile mitochondria at treated with either 2 μM scrambled A$\beta_{1\text{-}42}$ or A$\beta_{1\text{-}42}$ oligomers for 1 h before imaging with or without directionality over 150 s of live imaging in Day 70-95 iPSC-derived cortical neurons from the Exon 4 isogenic panel. Mean ± SEM. *n* = 8 (*MAPT+/+* scrambled A$\beta_{1\text{-}42}$), 13 (*MAPT+/+* A$\beta_{1\text{-}42}$), 6-9 (*MAPT-/-* scrambled A$\beta_{1\text{-}42}$) and 12-14 (*MAPT-/-* A$\beta_{1\text{-}42}$) microfluidic chambers measured across five independent neuronal differentiation repeats. Two-tailed Mann-Whitney test was used for statistical analysis.

(C) Quantifications of relative mitochondrial membrane potential (ψm) measured with JC-10 dye in Day 52-56 iPSC-derived cortical neurons from both isogenic panels at baseline compared to the *MAPT+/+* line. Mean ± SEM. *n* = three independent neuronal differentiation repeats. Kruskal-Wallis test with Dunn's multiple comparison correction was used for statistical analysis.

(D) Quantifications of relative mitochondrial membrane potential (ψm) measured with JC-10 dye in Day 52-56 iPSC-derived cortical neurons from both isogenic panels treated with either 2 μM scrambled A$\beta_{1\text{-}42}$ or A$\beta_{1\text{-}42}$ oligomers for 1 h before imaging compared to the *MAPT+/+* line. Mean ± SEM. *n* = three independent neuronal differentiation repeats. Kruskal-Wallis test with Dunn's multiple comparison correction was used for statistical analysis.

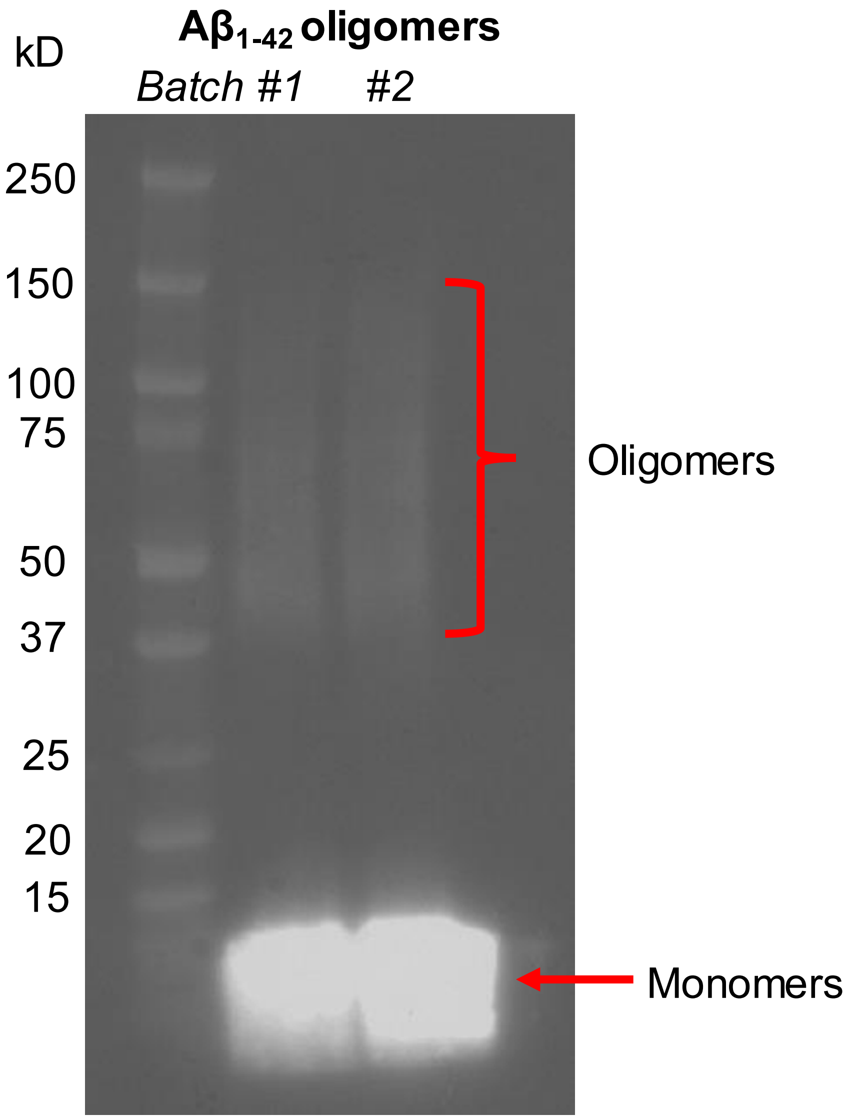

Supplementary Fig. 9

Supplementary Figure 9: Aβ_{1-42} oligomerisation validation.

Western blot of two independently oligomerised batches of 9 μg Aβ_{1-42} probed by 6E10 antibody. The arrow and bracket indicate the presence of monomers and a smear of oligomers between 40-150 kD (approximately 9-mer to 33-mer) in our oligomer samples treated to the iPSC-derived cortical neurons.

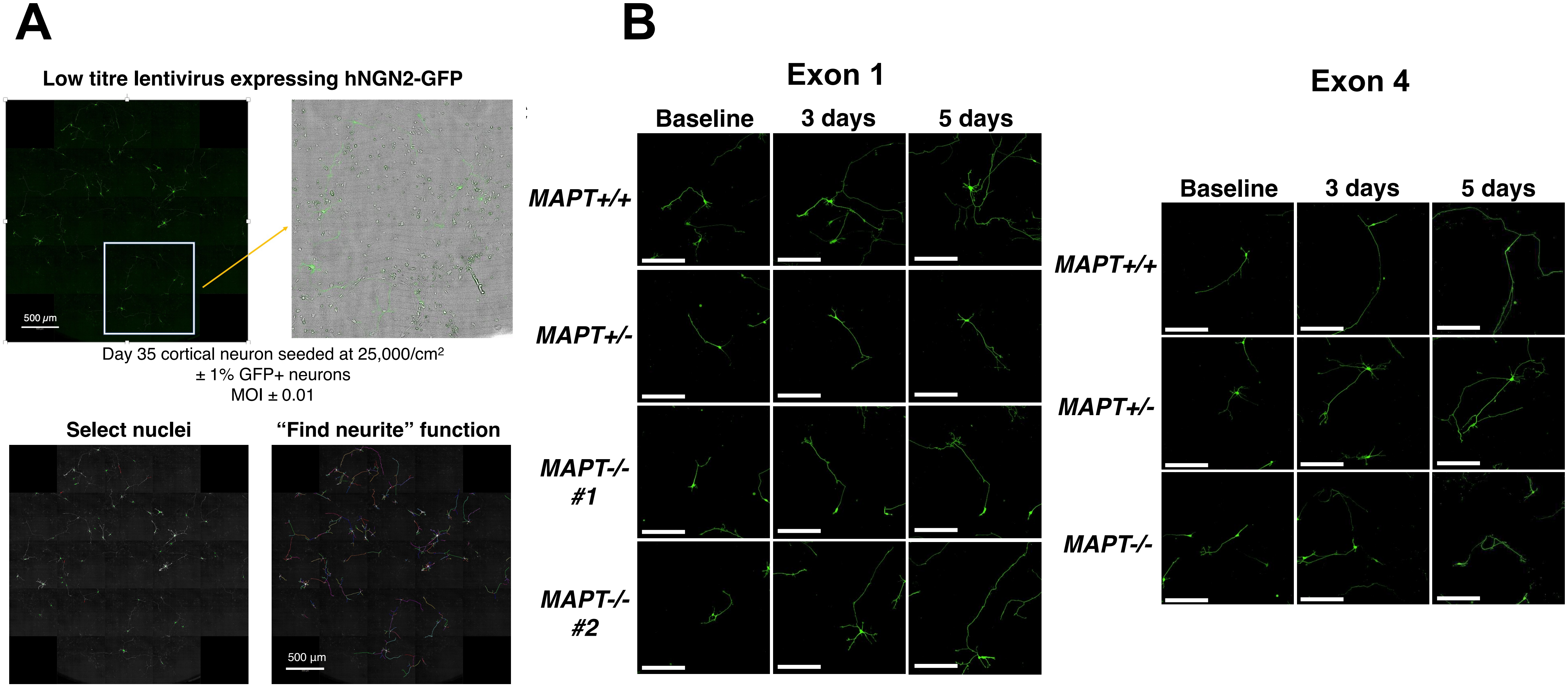

Supplementary Fig. 10

C

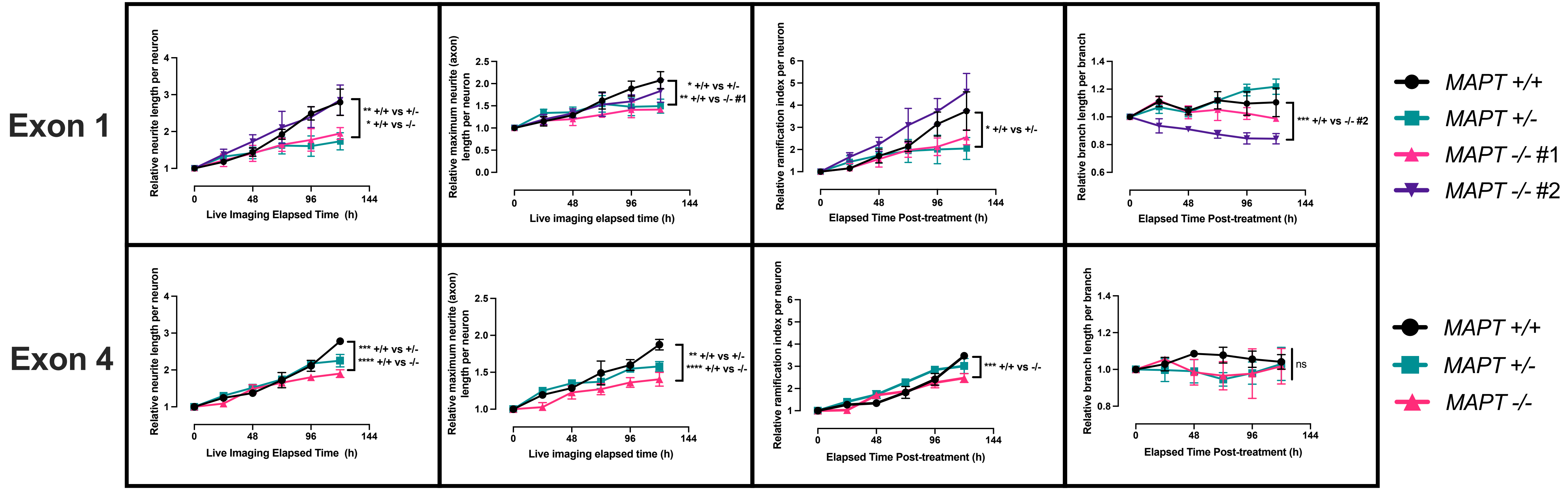

Supplementary Fig. 10

Supplementary Figure 10: Neurite outgrowth impairment in *MAPT-/-* neurons except the Exon 1 *MAPT-/-* #2 line.

(A) Schematic of neurite tracing experiment design. The images show a subset of Day 35 iPSC-derived cortical neurons expressing GFP, and in the Harmony® software analysis pipeline identifying GFP-positive nuclei and neurites stemming from those nuclei. Scale bar = 500 µm.

(B) Representative fluorescent images of Day 35 iPSC-derived cortical neurons in both isogenic panels demonstrating neurite outgrowth over 5 days of live imaging. Scale bar = 100 µm.

(C) Quantifications of relative neurite outgrowth, axonal outgrowth, ramification index and neurite branch length in Day 35 iPSC-derived cortical neurons over 5 days of live imaging compared to the baseline (i.e., elapse time = 0 h). Mean ± SEM. *n* = three independent neuronal differentiation repeats. Two-way ANOVA with Dunnett's multiple comparison correction was used for statistical analysis.

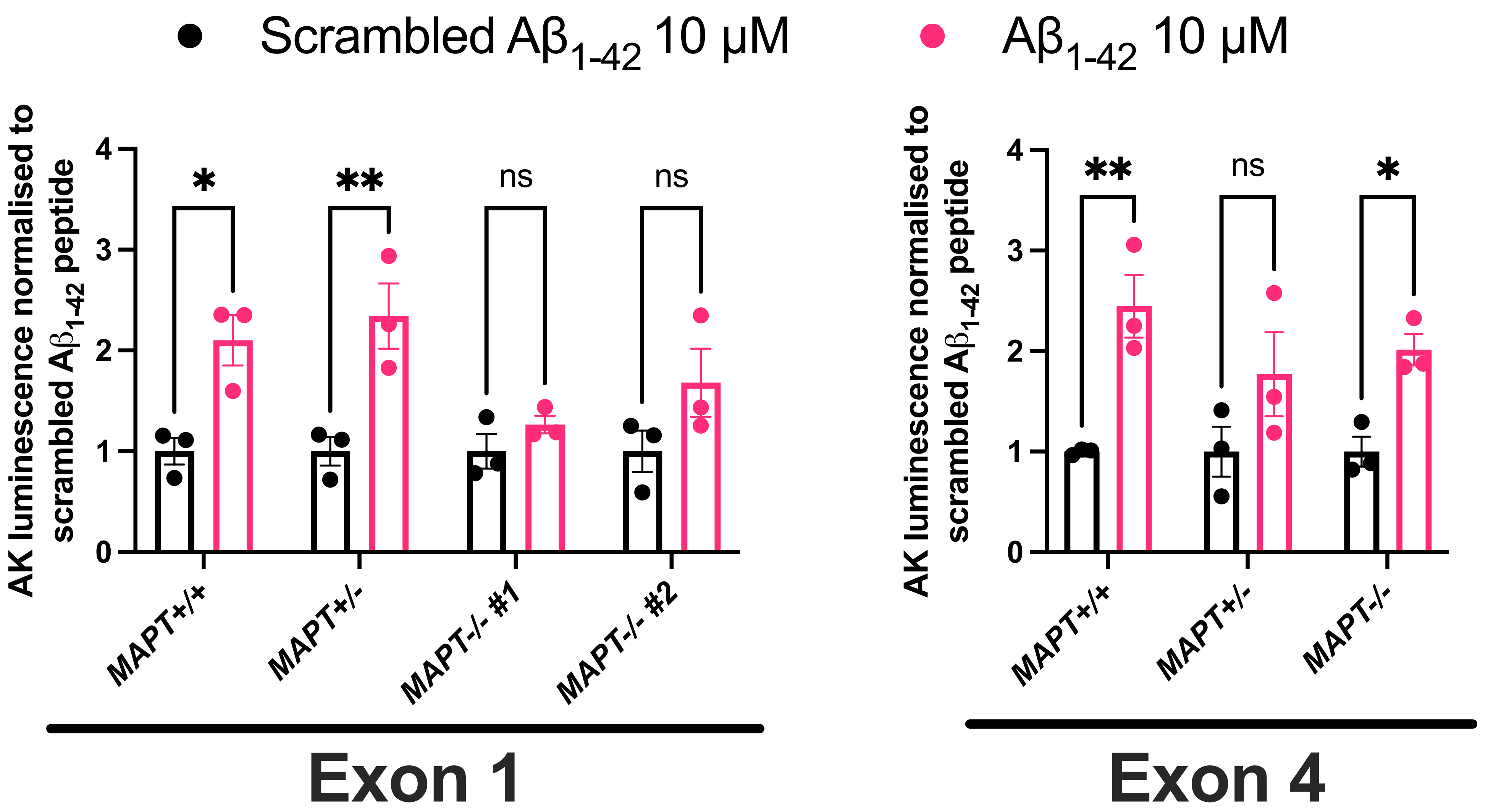

Supplementary Fig. 11

Supplementary Figure 11: Adenylate kinase-based cell viability assay on Day 79-86 iPSC-derived cortical neurons from both *MAPT-/-* isogenic panels.

The neurons were treated with 10 μM Aβ_{1-42} oligomers and scrambled control for 5 days. Bar graphs depicting relative adenylate kinase luminescence to the scrambled Aβ_{1-42} peptide. Mean ± SEM. N = three independent cortical neuron differentiation repeats. Two-way ANOVA with Šídák's multiple comparison test.

Supplementary Methods and Materials

All reagents were purchased from Sigma-Aldrich (Merck), and all cell cultures were maintained at 37°C with 5% CO_2 in a humidified incubator unless stated otherwise.

Identification of two panels of isogenic MAPT-/- iPSC lines

Genomic DNA was extracted from each iPSC colony using a commercial kit (Qiagen), before the DNA samples were amplified using the BigDye™ v3.1 reagent and sequenced with primers targeting either Exon 1 or Exon 4 spanning the gRNA binding sites (Supplementary Table 1). Before sending the DNA samples (10 μL per iPSC clone) for sequencing, samples were precipitated by adding 2 μL of 125 mM EDTA, 2 μL of 3 M sodium acetate and 50 μL of 100% ethanol to each sample. The samples were briefly vortexed before centrifugation at 3,000 g at 4°C for 30 min. After removing the supernatant by centrifugation at 185 g for 15 s, the DNA precipitates were washed with 70 μL of 70% ethanol per sample.

The sequencing results were analysed with the SnapGene® Viewer v5.2.4 and BLAST® (1) to identify candidate iPSC clones that underwent successful genetic modifications at the targeted regions. These candidate iPSC clones were further sub-cloned into pGEM®-T Easy Vectors (Promega) to determine the genetic modifications at the allelic level using the same primers for the Exon 1 and 4 targeted regions. The vectors were then transformed into One Shot™ TOP10 Chemically Competent *E. coli* (Thermo) by heat shock at 42°C for 45 s. At least six bacterial clones were picked and sequenced the way it was described above using T7 and SP6 promoter primers (embedded in pGEM®-T Easy Vectors).

Once the homozygosity/heterozygosity of the intended genetic modifications was determined, the sequence-confirmed iPSC clones were differentiated into neural progenitor cells (NPCs) or neurons and their cell lysates were analysed by western blot to confirm tau depletion.

Lentivirus generation

The lentiviral particles used in this study expressing genes of interest were generated by expressing lentiviral proteins in HEK293T cells grown in DMEM/F12 medium supplemented with 10% FBS, 2 mM L-glutamine, 100 μM non-essential amino acids and 50 U/ml Penicillin-Streptomycin (all from Thermo). HEK293T cells were passaged with TrypLE™ (Thermo) and seeded at 80,000 cells/cm^2 (1,000,000 cells in a T75 flask) 24 h before plasmid transfection.

The following protocol describes the reagents used for a single T75 flask of HEK293T cells. A Lipofectamine™ 3000 transfection reagent (Thermo) was used to deliver plasmids into HEK293T cells. 40 μL Lipofectamine™ 3000 transfection reagent was first mixed with 50 μL of P3000 reagent (from the same kit) in 1 mL of Opti-MEM™ medium (Thermo) for at least 5 min. Second-generation lentiviral packaging plasmids psPAX2 (#12260; 10 μg) and pMD2.G (#12259; 5.65 μg) were mixed with plasmids expressing genes of interest (FUdeltaGW-rtTA (rtTA; #19780; 9.6 μg), pTet-O-Ngn2-puro (Ngn2; #52047; 9.6 μg) or pLV-TetO-hNGN2-eGFP-Puro (Ngn2-GFP; #79823; 9.6 μg)) to achieve equimolar ratios in 1 mL of Opti-MEM™ medium in another tube. All plasmids were purchased from Addgene. Finally, the plasmid tube was added to the Lipofectamine tube dropwise before adding the mixture to the cells now grown in 13 mL of the above-mentioned medium without Penicillin-Streptomycin. The

transfection reagents were left overnight and replaced with fresh medium the following day with Penicillin-Streptomycin.

The lentivirus-containing cell supernatant was collected 48 h after the last medium change, and it was centrifuged at 400 g for 5 min to remove any cells before filtering through a cellulose acetate membrane with 0.45 μm pore size (Millipore). The supernatant was snap-frozen in dry ice before storing it at -80°C. For the pLV-TetO-hNGN2-eGFP-Puro virus, the aliquots were thawed once and sub-aliquoted in small volume to keep the lentivirus titre consistent since a small amount of this lentivirus was needed per transduction.

Differentiation of iPSC lines into cortical neurons

All iPSC lines were seeded on Matrigel® (Corning)-coated plates and cultured in mTeSR™ medium (STEMCELL Technologies) which was replaced daily. Fully confluent iPSC culture was passaged at a 1:6 ratio with TrypLE™ (Thermo) for 5 min in the incubator and pelleted by centrifugation at 400 g for 5 min.

Two variations of cortical neuron differentiation protocols were adapted and combined from Shi *et al.* (pharmacologically directed) (2) and Zhang *et al.* (transcription factor directed) (3) into a single protocol and adapted as previously described (4). All media were filtered through a polyethersulfone membrane with a 0.22 μm pore size (Millipore). All cortical neuron cultures were seeded in co-culture with a confluent layer of primary rat cortical astrocytes (Thermo) except for harvesting cell lysate or live assays with dyes which are not cell-type specific. For the neuronal monoculture without rat astrocytes, the end point of differentiation was set to be Day 50 or before to ensure

that the culture remained relatively homogeneous without any contamination with non-neuronal cells that might emerge sporadically throughout the differentiation process.

The first 25 days of cortical neuron differentiation was adapted from Shi et al. (2). All iPSC lines were first seeded concurrently on Geltrex™ (Thermo)-coated plates at 105,000 cells/cm^2 (1,000,000 cells in a well of a six-well plate) in mTeSR™ medium supplemented with 10 μM Y-27632 (Tocris). The Y-27632 compound was removed from the medium the next day and subsequently the medium was replaced daily. When the iPSC culture became confluent (Day 0), it was washed once with phosphate-buffered saline (PBS) before changing to neural induction medium (NIM) which is neural maintenance medium (NMM) supplemented with 10 μM SB-431542 (Tocris) and 1 μM Dorsomorphin. NMM contains two types of media at 1:1 ratio: (1) Neurobasal™ medium (Thermo) supplemented with 1X B-27™ supplement (Thermo), 25 U/ml Penicillin-Streptomycin (Thermo) and 1 X GlutaMAX (Thermo) (2) DMEM/F12 medium (Thermo) supplemented with 1X N-2 supplement (Thermo), 50 μM non-essential amino acid (Thermo), 50 μM 2-mercaptoethanol (Thermo), 2.5 μg/ml insulin, 1 mM sodium pyruvate (Thermo) and 2 mM L-glutamine (Thermo). The NIM was subsequently replaced daily until Day 12.

The cells were passaged on Day 12 with 1 mg/ml dispase (10 mg/ml dispase dissolved in PBS and added directly to the cell culture media), for approximately 10 to 30 min in the incubator depending on the lifting efficiency. A sheet of cells would detach from the plate bottom and was then broken up by pipetting once before transferring to 10 mL of NIM. The cell aggregates were allowed to settle by gravity before they were

washed by 10 mL of NIM twice. Finally, the cell aggregates were seeded onto 15 μg/ml fibronectin-coated plates at a 1:2 ratio in NIM.

On Day 13 and 15, the cells were provided with NMM supplemented with 20 ng/ml FGF2. On Day 17, the cell cultures were passaged as on Day 12 except that the cells were washed and plated in NMM. The cell aggregates were seeded onto 100 μg/ml poly-L-ornithine plus 10 μg/ml laminin (PO/L)-coated plates at a 1:3 ratio (the plates were first coated with PO overnight at 37°C, before they were washed once with PBS and then coated with laminin overnight). Finally, the NMM was replaced every two days until Day 25 where the NPCs were lifted by incubating with Accutase® and cryo-preserved at 2,000,000 cells/vial in NMM supplemented with 20 ng/ml FGF2 and 10% dimethyl sulfoxide.

The second part of the differentiation protocol beyond Day 25 was modified from Zhang *et al.* (3) and the protocol relies on a doxycycline-inducible overexpression system of *Ngn2* to result in an excitatory cortical neuronal identity. Each vial of NPCs was thawed, spun down, and plated into a single well in a twelve-well plate (Day 25). *Ngn2* and *rtTA* lentiviruses (25 μL each) were added into the cell suspension in NMM (supplemented with 20 ng/ml FGF2 and 10 μM Y-27632) as the NPCs settled onto PO/L-coated plates. The medium was replaced 8 h later with fresh NMM. On Day 28, the NMM was replaced by neuronal medium consisting of Neurobasal™ medium (Thermo) supplemented with 1X B27™ (Thermo), 1X GlutaMAX™ (Thermo), 25 U/ml Penicillin-Streptomycin (Thermo), 10 ng/ml neurotrophin-3, 10 ng/ml BDNF (Peprotech), 1 μg/ml doxycycline, 200 ng/ml laminin and 200 μM ascorbic acid. On Day 30, the neuronal medium was replaced and supplemented with 1 μg/ml puromycin.

On Day 32, the neurons were passaged with Accutase® and seeded in co-culture with a confluent monolayer of primary rat cortical astrocyte seeded onto 10 μg/ml poly-D-lysine-coated plates (coated overnight and washed once with PBS before seeding) at its third passage (Thermo). The iPSC-derived neuron-rat astrocyte co-culture was subsequently half-fed with the neuronal medium only twice a week. On Day 40, the co-culture was treated with 100 nM Ara-C which was gradually removed over subsequent half-feedings. The neuronal culture was then allowed to age until Day 80 or after for downstream experiments.

When co-culture was not suitable for a particular experiment, the neurons were seeded onto PO/L-coated plates. The primary rat cortical astrocyte culture was maintained in DMEM with high glucose supplemented with 15% foetal bovine serum (FBS) (Thermo), 2 mM L-glutamine (Thermo) and 50 U/ml Penicillin-Streptomycin (Thermo). After the neurons were seeded onto the astrocyte, the co-culture was maintained in the neuronal medium only.

Reverse-transcription PCR (RT-PCR)

Day 35 NPCs were harvested for RNA extraction in buffer RLT with β-mercaptoethanol and the RNA was extracted using RNeasy kit according to the manufacturer's protocol (Qiagen). It was then converted into cDNA using SuperScript III (ThermoFisher Scientific) as instructed by manufacturer. The expression of *MAPT* was measured using RT-PCR (GoTaq Hot Start, Promega), according to the manufacturer's protocol. The forward primer was placed in Exon 0: 3' CCTCGCCTCTGTCGACTATC 5', and the reverse primer in Exon 11: 3'TGGTTTATGATGGATGTTGCCTAA 5'. The product was loaded onto a 1.5% agarose gel for electrophoresis before visualisation.

Quantitative reverse-transcription PCR (qRT-PCR)

RNA was extracted using TRIzol extraction (ThermoFisher Scientific) according to the manufacturers protocol. It was then converted into cDNA using SuperScript III (ThermoFisher Scientific) as instructed by manufacturer. The expression of specific mRNAs was measured using qRT-PCR (Fast-SYBR, Applied Biosystems). The samples were loaded in triplicates and the qRT-PCR was performed using the StepOnePlus software. They were analysed using the delta delta Ct method. *GAPDH* was used as a housekeeping gene. The primer were designed to probe for different exons within the *MAPT* gene and are all detailed in Supplementary Table 2.

Long-read sequencing and analysis

RNA samples from Day 35 NPCs from the *MAPT* Exon 4 isogenic panel were sequenced using the Oxford Nanopore Technologies (ONT) MinION platform. The library preparation was performed using the cDNA-PCR sequence specific kit (SQK-PCS111), for which the following primer was used: 5' ACTTGCCTGTCGCTCTATCTTC GGGATATCTCTAACCACC 3'. The first 22 base pairs are a specific nanopore sequence while the following base pairs target *MAPT* exon 11. Base calling of the reads was performed using the ONT Dorado software (v0.5.0) and read data from the FASTQ files were then aligned to the human genome (GENCODE M25 GRCh38.p7) using minimap2 (v2.26) (5). The Integrative Genomics Viewer (IGV) genome browser was used to analyse the reads (IGV 2.16.2, GRCh39/hg19, https://www.igv.org/) (6).

Western blot

The NPCs were seeded on PO/L-coated plates on Day 25 and maintained until Day 30 for harvesting without virus transduction, while others were transduced with the *Ngn2* lentivirus and final-plated on Day 32 for harvesting on Day 50 as neurons. The cells were washed once with PBS before they were lysed on ice in radioimmunoprecipitation assay (RIPA) buffer containing 50 mM Tris-HCl, 150 mM NaCl, 1% (v/v) Triton X-100, 1% (w/v) sodium deoxycholate and 0.1% (w/v) sodium dodecyl sulphate (SDS) at pH 7.4. The RIPA buffer was supplemented with cOmplete™ mini EDTA-free protease inhibitor cocktail (Roche). The lysate was then transferred into a tube on ice and left incubating for at least 15 min. Finally, the lysate was centrifuged at 20,000 g for 30 min at 4°C before the supernatant was frozen at -80°C.

To quantify protein concentration in the lysates, a Pierce™ Bicinchoninic Acid Protein Assay Kit (Thermo) was used following manufacturer's instructions. Briefly, 12.5 μL of either bovine serum albumin (BSA) standards or cell lysates were added into a 96-well plate followed by 100 μL of the BCA reagent. The plate underwent incubation at 37°C for 30 min before it was read for absorbance at 562 nm wavelength in a PHERAstar® microplate reader (BMG Labtech). Protein concentration was determined by comparing to a BSA standard curve.

For western blots to examine total tau protein, cell lysates underwent dephosphorylation with (final concentration in the mixture) 10% $MnCl_2$, 10% reaction buffer and 0.38 μL lamda-phosphatase (New England Biolab; sufficient for up to 10 μg of cell lysate) for 1 h at 30°C prior to adding the sample loading dye for denaturation.

Cell lysates were then denatured by heating at 95°C for 10 min with sample loading dye containing (final concentration in the mixture) 2% SDS, 5% 2-mercaptoethanol, 10% glycerol, 0.002% bromophenol blue and 375 mM Tris at pH 6.8. For the western blot probing for oligomeric amyloid-$\beta_{1\text{-}42}$ (A$\beta_{1\text{-}42}$), the reducing agent 2-mercaptoethanol was omitted on purpose for the signals to show (Supplementary Fig. 9).

Denatured samples were loaded into precast 4-15% gradient polyacrylamide gels (Bio-Rad) submerged in running buffer consisting of 25 mM Tris base, 192 mM glycine and 0.1% SDS. The gel was subject to 100 V for 3.5 h and the gel underwent semi-dry transfer by a Trans-Blot® Turbo™ Transfer System (Bio-Rad) onto a 0.2 μm polyvinylidene fluoride membrane at 1.3 A for 11 min. The resultant blot was washed once in tris-buffered saline with 0.1% Tween-20 (TBST) and blocked in 5% milk dissolved in TBST shaking for 1 h at room temperature. The blot was then incubated with primary antibodies (Supplementary Table 3) diluted in 1% milk in TBST rocking overnight at 4°C.

The blot was washed with TBST shaking for 10 min at room temperature three times prior to incubation with secondary antibodies (Supplementary Table 3) diluted in 1% milk in TBST for 1 h at room temperature. Thereafter, the blot was washed for for three more times before adding a Immobilon® Western Chemiluminescent HRP Substrate (Millipore) to the blot. The blot was imaged in the ChemiDoc™ Imager (Bio-Rad) and analysed in the Image Lab v6.1 (Bio-Rad) software.

Immunoprecipitation-mass spectrometry (IP-MS) analysis

All *MAPT+/+* and *MAPT-/-* iPSC lines from both isogenic panels were differentiated into Day 50 neurons but every line was maintained separately to ensure no risk of cross-contamination. Cells were washed with PBS once and lysed by adding RIPA buffer supplemented with cOmplete^{TM} mini EDTA-free protease inhibitor cocktail (Roche) directly into the wells followed by incubation on ice for 30 mins. Following centrifugation at 21,300 g for 30 min at 4°C to remove cellular debris, the supernatant was stored at -80°C until further processing. Protein concentration was measured with the Pierce™ BCA Protein Assay Kit (Thermo) following manufacturer's instructions. To enable the detection and quantification of low levels of tau, total tau was enriched by IP with the polyclonal K9JA anti-tau antibody clone (Dako, cat. no. A0024, Lot No. 20043728) from 400 µg protein lysate for the Exon 1 *MAPT+/+* and *MAPT-/-* #2 lines, 200 µg protein lysate for the Exon 1 *MAPT-/-* #1 line and 450 µg protein lysate for the Exon 4 *MAPT+/+* and *MAPT-/-* lines. For this, protein lysates were incubated in low-protein binding tubes with the antibody (20 µg antibody/mg protein lysate) overnight at 4°C with end-over-end rotation (7 rpm). The antibody-protein complexes were then captured by addition of Protein G magnetic Dynabeads (5 µL beads/µg antibody; Thermo Scientific, cat. no. 13424229) followed by further incubation for 1 h at 4°C with end-over-end rotation. Flow-through containing unbound proteins was removed and beads were washed three times with TBST. Beads were then transferred to fresh low-protein binding tubes and antibody-protein complexes were eluted by incubating in 60 µL 1x Laemmli Sample Buffer (Biorad, cat. no. 1610747, reducing agent was omitted). Eluates were snap-frozen on dry ice and stored at -80°C.

Proteins present in eluates were identified by liquid chromatography-MS/MS analysis. Samples were measured using a Dionex Ultimate 3000 nano-ultra high pressure reversed-phase chromatography system connected to an Orbitrap Fusion Lumos mass spectrometer (Thermo). Peptides were separated by an EASY-Spray PepMap RSLC C18 column (500 mm x 75 µm, 2 µm particle size; Thermo) over a 60 min gradient of 2-35% acetonitrile in 5% DMSO, 0.1% formic acid and at a flow rate of 250 nL/min. The column temperature was maintained at 50°C. The mass spectrometer was operated in positive polarity mode with a capillary temperature of 275°C. Data-independent acquisition mode was utilized for automated switching between MS and MS/MS acquisition, as previously described (7). Approximately 200 ng of peptide material was loaded on column. Samples from the *MAPT-/-* lines were run on the mass spectrometer before those from the *MAPT+/+* lines to eliminate any risk of carry-over. As an additional precaution, blank samples were run on the MS instrument before the eluates from the *MAPT-/-* lines. Data was searched using data-independent acquisition-neural network (version 1.8) against a *Homo sapiens* Uniprot database (retrieved on 16/04/2021), using default settings. All MS data was deposited in Proteome Xchange (PRIDE) and can be accessed using the unique identifier PXD038918.

Extraction of human brain homogenate

The AD post-mortem brain homogenate extraction protocol was modified from Jin *et al*. (8). To elaborate, 1 g of AD cortical tissue was thawed on ice prior to homogenisation in a Dounce homogeniser for 25 strokes in 4 mL of cold artificial CSF (aCSF; 124 mM NaCl, 2.8 mM KCl, 1.25 mM NaH_2PO_4 and 26 mM $NaHCO_3$, pH = 7.4) supplemented with a panel of protease inhibitors (5 mM EDTA, 1 mM EGTA, 5 ug/ml

leupeptin, 5 µg/ml aprotinin. 2 µg/ml pepstatin, 120 µg/ml Pefabloc and 5 mM NaF). The homogenised mixture then underwent ultracentrifugation at 200,000 g for 110 min at 4°C with a SW-41 Ti rotor (Beckman Coulter) and transferred the supernatant into a Slide-A-Lyzer™ G2 Dialysis Cassettes 2K MWCO (Thermo) in a 100X volume of aCSF without the protease inhibitors swirling for 72 h. The aCSF bath was replaced every 24 h. The resultant brain homogenate was frozen at -80°C.

The AD cortical tissues were obtained from the Oxford Brain Bank. Whole temporal cortices from an AD patient and healthy control (73 years old, female, Braak stage VI, 75 h delay post-mortem, *APOE* ε3/ε3; 70 years old female healthy control, Braak stage I, 81 h delay post-mortem, *APOE* ε3/ε3) were used for this study. In addition, approximately 4 g each of frontal cortices from two AD patients (#1: The same 73-year-old female and #2: 81 years old, male, Braak stage VI, 26 h post-mortem delay, *APOE* ε4/ε4) were used to enrich for AD brain-derived Aβ. The brain homogenates from both frontal cortices were pooled together for experiments.

Immunodepletion and enrichment of AD brain-derived Aβ

Immunodepleted (ID) controls were included in a subset of the experiments using AD brain homogenate as Aβ insult. First, Protein A (if the antibody host was rabbit) or Protein G (if the antibody host was mouse) agarose beads (Abcam) were washed three times in aCSF and centrifuged at 400 g for 5 min at 4°C. The beads were then resuspended in 50% slurry with aCSF. At the same time, the brain homogenate aliquots were thawed on ice and centrifuged at 16,000 g at 4°C for 2 min to remove any insoluble component. The agarose beads were then added to the brain homogenate at 3% v/v. Thereafter, 3 mg/ml of S97 anti-Aβ antibody (extracted from

a vial of rabbit serum provided by Professor Dominic Walsh; host species: rabbit) at 1:100 dilution (for immunodepletion) or 3 μg/ml of each 4G8 and 6E10 anti-Aβ antibodies (Biolegend) (for brain-derived Aβ enrichment) were added to the brain homogenate-agarose beads mixture. Mock immunodepletion was performed on the AD and healthy control brain homogenates with normal rabbit or mouse IgG antibodies (Abcam).

The mixture was left rotating at 4°C for 12 h before it was centrifuged at 400 g for 5 min at 4°C. The supernatant was transferred for another two rounds of mock or actual immunodepletion whereas the beads were kept in PBS at 4°C for the brain-derived Aβ elution process later. Finally, the supernatant was mixed with Protein A or G agarose beads only (at 2% v/v) for 2 h before the brain homogenate was used for treatments on iPSC-derived cortical neurons.

The antibody-coated agarose beads that pulled down Aβ were washed 3 times with 1 ml of PBS, each time settling the beads by centrifugation at 400 g for 5 min at 4°C. The beads-bound Aβ was then eluted by adding 0.1 M glycine (pH 2.8) that was equivalent to 3 times in volume of the beads. The tube was left rotating at room temperature for 10 min and the eluate was transferred to an equal volume of 1 M Tris (pH 8.0). This elution step was repeated for another two times on the same beads before the eluate was transferred to a centrifuge filter protein concentrator (3 kDa molecular weight cut-off). The concentrators underwent centrifugation at 14,000 g for 2 h at 4°C and the Aβ solution that remained above the filter was diluted in PBS before storing at -80°C.

Soluble Aβ quantification assay

The levels of Aβ in the brain homogenates were quantified using a V-Plex Plus Aβ Peptide Panel 1 (6E10) Kit from Meso Scale Discovery according to the manufacturer's instructions. The samples of interest were diluted 1 in 2 in the assay buffer for quantification. The Meso Scale Discovery Workbench 4.0 software was used to analyse Aβ levels.

The AD brain-derived Aβ levels were quantified using a Cisbio $A\beta_{1\text{-}40}$ kit. The Aβ levels were detected via homogeneous time-resolved fluorescence from a pair of antibodies. The samples were diluted 1 in 8 for incubation overnight at 4°C. The plate was then read in a PHERAstar® microplate reader (BMG Labtech) to detect fluorescence signals at wavelengths 665 and 620 nm. The data were represented as ΔF which is a relative value to the 665/620 nm signal ratio of the negative control.

Oligomerisation of synthetic $A\beta_{1\text{-}42}$ peptides

Both lyophilised $A\beta_{1\text{-}42}$ and treatment control scrambled $A\beta_{1\text{-}42}$ peptides (Bachem, H-1368 and H-7406) were resuspended to 1 mM in Hexafluoro-2-propanol (HFIP). The tubes were vortexed and left sitting at room temperature for 30 min, before they were aliquoted and dried in a Speed-Vac concentrator for 30 min (Thermo) and stored at -80°C. To oligomerise the $A\beta_{1\text{-}42}$ peptides, the Aβ film was first resuspended in dimethyl sulfoxide to 5 mM before it was sonicated in a water bath at room temperature for 10 min. The peptides were subsequently diluted to 100 μM in PBS and the tubes were left stationary at 4°C for 24 h. Just before treating the cells with Aβ oligomers, the

solution was centrifuged at 14,000 g for 10 min at 4°C to remove any precipitate/fibrils. The $A\beta_{1-42}$ oligomers were used at a concentration range from 2 to 10 µM.

Immunocytochemistry

Adherent neurons were fixed in 4% paraformaldehyde for 5 min (for synaptic staining) or 20 min (for other markers), followed by treating with 0.05% saponin in PBS for 20 min for permeabilisation. To block the samples, the plates were treated with 10% normal goat serum (NGS) with 0.01% tween-20 in PBS for 30 min. Primary antibodies were then left incubating with the samples at 4°C overnight with 1% NGS and 0.01% tween-20, before washing 3 times with PBS. Secondary antibodies were then applied in 1% NGS and 0.01% tween-20 at room temperature for 1 h before washing for another 4 times before imaging on the same day. The primary and secondary antibodies used are listed in Supplementary Table 3.

References

1. Altschul SF, Gish W, Miller W, Myers EW, Lipman DJ. Basic local alignment search tool. J Mol Biol. 1990 Oct 5;215(3):403–10.
2. Shi Y, Kirwan P, Livesey FJ. Directed differentiation of human pluripotent stem cells to cerebral cortex neurons and neural networks. Nat Protoc. 2012;7(10):1836–46.
3. Zhang Y, Pak CH, Han Y, Ahlenius H, Zhang Z, Chanda S, et al. Rapid single-step induction of functional neurons from human pluripotent stem cells. Neuron. 2013 Jun 5;78(5):785–98.
4. Hedegaard A, Monzón-Sandoval J, Newey SE, Whiteley ES, Webber C, Akerman CJ. Pro-maturational Effects of Human iPSC-Derived Cortical Astrocytes upon iPSC-Derived Cortical Neurons. Stem Cell Reports. 2020 Jul 14;15(1):38–51.
5. Li H. Minimap2: pairwise alignment for nucleotide sequences. Bioinformatics. 2018 Sep 15;34(18):3094–100.
6. Robinson JT, Thorvaldsdóttir H, Winckler W, Guttman M, Lander ES, Getz G, et al. Integrative genomics viewer. Nat Biotechnol. 2011 Jan;29(1):24–6.
7. Demichev V, Messner CB, Vernardis SI, Lilley KS, Ralser M. DIA-NN: neural networks and interference correction enable deep proteome coverage in high throughput. Nat Methods. 2020 Jan 1;17(1):41–4.
8. Jin M, O'Nuallain B, Hong W, Boyd J, Lagomarsino VN, O'Malley TT, et al. An in vitro paradigm to assess potential anti-Aβ antibodies for Alzheimer's disease. Nat Commun. 2018;9(1).
